# Hidden challenges behind ecosystem services improvement claims

**DOI:** 10.1016/j.isci.2022.104928

**Published:** 2022-08-13

**Authors:** Qing Yang, Gengyuan Liu, Linyu Xu, Sergio Ulgiati, Marco Casazza, Yan Hao, Zhongming Lu, Xiaoya Deng, Zhifeng Yang

**Affiliations:** 1 Key Laboratory for City Cluster Environmental Safety and Green Development of the Ministry of Education, School of Ecology, Environment and Resources, Guangdong University of Technology, Guangzhou 510006, China; 2 Advanced Interdisciplinary Institute of Environment and Ecology, Beijing Normal University, Zhuhai 519087, China; 3 Southern Marine Science and Engineering Guangdong Laboratory (Guangzhou), Guangzhou 511458, China; 4 State Key Joint Laboratory of Environmental Simulation and Pollution Control, School of Environment, Beijing Normal University, Beijing 100875, China; 5 Department of Science and Technology, University of Naples ‘Parthenope’, Centro Direzionale, Isola C4, 80143 Naples, Italy; 6 Department of Medicine, Surgery and Dentistry, University of Salerno, Baronissi, Salerno, Italy; 7 Division of Environment and Sustainability, Hong Kong University of Science and Technology, Clear Water Bay, Kowloon, Hong Kong SAR, China; 8 State Key Laboratory of Simulation and Regulation of Water Cycle in River Basin, Department of Water Resources, China Institute of Water Resources and Hydropower Research, Beijing 100038, China

**Keywords:** Environmental management, Environmental monitoring, Environmental policy, Natural resources

## Abstract

Substantial evidence indicates that China’s afforestation statistically contributed to the ecosystem services (ES) improvement. However, we found the potential challenges behind this improvement, especially in water-limited areas. We propose an attribution analysis method, which can assess the specific contribution of natural, human and cognition degree drivers to ES dynamics. The results found that the ratio of natural and human drivers in the area north of China’s 400 mm precipitation isopleth is 2:7. This means local vegetation capacity has already exceeded water limitation, implying a conflict between nature and humans. However, the natural contribution in the area between 400 and 800 mm precipitation isopleth is negative, whereas the human contribution is 91%. This means this area has fragile natural conditions and needs more flexible policies. The ratio of natural and human drivers in the region south of 800 mm precipitation isopleth is 6:3, suggesting the ecological policies here can be maintained.

## Highlights

• PDE is used to assess the contribution of natural and human drivers to ES changes
• Land use management dominantly contributed (55%) to China’s ES improvement
• Re-vegetation in the area north of 400 mm precipitation isohyet exceeded NPP threshold
• The area between 400 and 800 mm isoprecipitation line is the key area with ES decrease

## Graphical abstract

**Figure:**
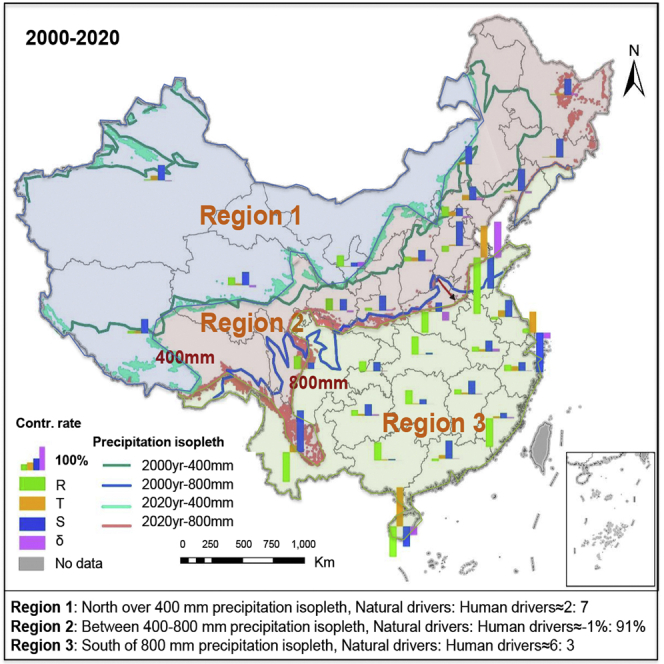

## Introduction

Ecosystem services (ES) are the benefits that humans obtain from ecosystems. Nearly two-thirds of ES are found to be declining globally (Millennium Ecosystem Assessment, 2005). To reverse this trend, China has implemented the world’s largest government-funded ecological programs: the Natural Forest Conservation Program (NFCP) and the Sloping Land Conversion Program (SLCP) (Liu et al., 2008; Zhang et al., 2000). A study indicated that these programs significantly contributed to the increase of China’s ES from 2000 to 2010 (Ouyang et al., 2016). Observational data (2000–2017) provided by the National Aeronautics and Space Administration (NASA) (Tabor, 2019) and another study (Chen et al., 2019) proved that China has achieved the largest world greening trend that is partly attributable (42%) to the implementation of forest conservation and expansion ecological programs, including the Three-North Shelterbelt Development Program (TNSDP), the Beijing-Tianjin Sand Source Control Program (BSSCP), NFCP, the Grain to Green Program (GTGP), and so on (Chen et al., 2019).

Besides the positive results, however, evidences suggest that the existing ES improvement plans, based on the expansion of vegetated areas, might generate undesirable pressures on resources, especially in relatively arid areas. For example, a work pointed out that tree-planting in Northwest China could lead to water resources depletion (Zastrow, 2019). In particular, the local arid climate conditions might aggravate the increase of absorbed precipitation and the reduction of surface runoff generated by an increase of vegetation. This, in turn, might have an adverse impact on human water demand (Fu et al., 2017). For example, the sustainable water resource limits were reached in China’s Loess Plateau because of ongoing re-vegetation projects (Feng et al., 2016). Considering the environmental constraints in this area, the permissible threshold of net primary productivity (NPP) was fixed at 400 ± 5gC m^−2^ yr^−1^ in the period 2000–2010; this value was already reached in year 2008 (Feng et al., 2016). Therefore, this study stated that crossing this threshold the water supply for the coupled anthropogenic-biological system might become unbalanced.

Despite the contrasting evidences, some arid and semi-arid regions in China plan to further implement such projects (China Gansu Net, 2020; FGBIMAR, 2020). For example, the 2020 work plan of the Inner Mongolia Forestry and Grass Bureau proposed to complete the afforestation of more than 20,000 ha (FGBIMAR, 2020). To avoid the risk of increasing impacts on a limited availability of resources, it is important to consider whether these policies can be beneficial for a balance between local ES improvement and resource limits. This problem should be solved through a quantitative spatial analysis, being able to assess the relation between greening plans and ES improvements, propose a zoning and classification for the management of ecosystems and their services according to local environmental conditions. This, in turn, relies on an appropriate method to quantify ES and their variations. However, quantitative analyses are missing in the case of ES improvement and the greening trends. Moreover, ES assessment methods are mainly based on monetary value, yet economic value of ES is a human perception-centered valuation and is not equal to market value, which cannot objectively assess an ecosystem’s contribution to human well-being because perceived value can be a quite limiting valuation criterion (Costanza et al., 2017).

The current methods of identifying the greening mechanisms mainly include deriving causality based on strong correlation (Ouyang et al., 2016), indicators based remote sensing observations and derived by models, such as Normalized Difference Vegetation Index (NDVI) (Goetz et al., 2005; Myneni et al., 1997; Piao et al., 2003), Leaf Area Index (LAI) (Chen et al., 2019; Zhu et al., 2016), and so on. For example, a study performed a correlation and regression analysis between the factors including the afforestation plans (i.e., SLCP, NFCP), the biophysical variables (such as above-ground forest biomass per unit area, as 10^3^tkm^−2^), the socioeconomic variables (e.g., simulating changes in human population density 2000 to 2010, as 10^3^ individual km^−2^), and ES improvement (Ouyang et al., 2016). The results suggested that China’s conservation policies significantly contributed (e.g.: SLCP (p< 0.01 or p< 0.05), NFCP (p< 0.001)) to increases in four key ES (carbon sequestration, soil retention, sandstorm prevention, and water retention) (Ouyang et al., 2016). Another research confirmed China’s greening as a consequence of its implementing ecological projects, such as TNSDP, BSSCP, NFCP, and GTGP (Chen et al., 2019). However, there still exist some challenges for the attribution analysis of the greening. Specifically, existing attribution analysis studies were mainly conducted through the correlation test between an area indicator (such as forest area, etc.) and economic variables, often misinterpreting a strong correlation as a causality; yet, correlation does not imply causation (Ksir and Hart, 2016). Second, the existing methods hardly distinguish the specific contributions of natural and human drivers (Hegerl et al., 2010; Piao et al., 2019). This is critical to determine the relative relevance between human and natural drivers in the observed ES improvement. Third, interactions and co-linearity effects among multiple factors are still poorly understood (Norby et al., 2010), leading to a difficulty in quantifying the individual contribution of each driver (Piao et al., 2019). As a consequence, ES assessments and future simulations are often unreliable.

A proper selection of ES accounting method is highly relevant to accurately identify the drivers of ES variations. Non-monetary methods express the flows of resources (i.e., materials, energy, and information) in biophysical units, and are more effective in considering ES under a broader social-ecological perspective, that puts ‘nature’ at the core (Costanza, 2020). Under a non-monetary framework, ES can be described as products of ecosystems whose functioning depends on a certain energy input (i.e., the annual input of solar energy and other biosphere driving forces coupled to human efforts). If the contribution of natural and human drivers can be separately identified in the procedure of ES accounting, it could be easier to determine the causal chain (if any) that affects the changes in ES.

Emergy accounting (EMA) method (Brown and Ulgiati, 2016; Odum, 1996), based on the calculation of the cumulative flows of available energy supporting the generation of a product or service, expressing in unit of solar equivalent joule (sej), has been widely applied non-monetary approach to account and simulate ES and natural capital (Brown et al., 2006; Campbell and Brown, 2012; Dong et al., 2012; Yang et al., 2018, 2019a, 2019b, 2020). From a donor-side perspective (i.e., on the accounting of direct and indirect inflows of resources), EMA can quantify the environmental workload supporting each flow or storage of resources. It can define the dynamics of a natural supply chain that generates the ES through a detailed analysis of material flows and energy transfer. It is also possible to distinguish the natural and human contributions, and assess the impacts on human health and ecosystem quality. Thus, the added value of the EMA method is that it looks at the processes of ES formation from the perspective of resource generation by biosphere, namely temporal and spatial scales needed by nature to make resources available for use by all species. This allows us to obtain a comprehensive quantification of scarcity and renewability of resources going beyond the existing monetary evaluations. However, this distinction could be improved, i.e., assessing the relative contribution of the different input factors through the application of partial differentiation equations (PDEs). In fact, this choice would avoid the problem of co-linearity of independent variables.

This study aims to propose an attribution analysis method for changes in ES based on ecological thermodynamics and partial differential equations, i.e., assess ES based on emergy method (Brown et al., 2006; Brown and Ulgiati, 2016; Campbell and Brown, 2012; Dong et al., 2012; Odum, 1996; Yang et al., 2018, 2019a, 2019b, 2020; STAR Methods) and quantify the specific contribution of drivers of ES variations based on PDEs (see STAR Methods). China’s ES dynamics over the past two decades and the specific contribution of natural and human drivers to the changes in ES in different geographic regions are quantitatively assessed. This study can provide policy recommendations on refined ecosystem management and conservation according to local conditions.

## Results

### China’s ES is increasing

**Figure 1:**
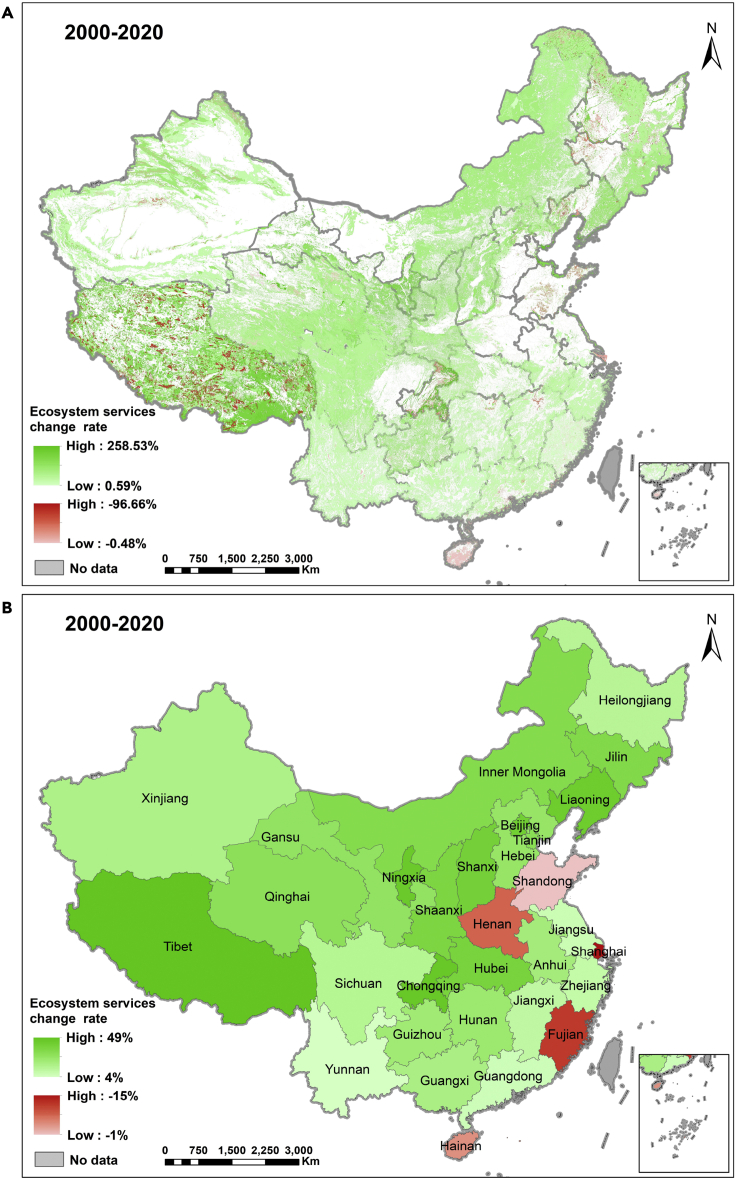
The change rate of China’s ecosystem services from 2000 to 2020 The green and red colors represent the increase and decrease in ecosystem services respectively; (A) indicates the change rate of ecosystem services of China’s different ecosystems; (B) shows the change rate of China’s provincial ecosystem services; the unit of ecosystem services is sej/yr.

Figures 1A and 1B and Table S4 show that ES increased in the period 2000–2020 in 89% of China’s ecosystem areas, whereas they declined for 11% of the same area. China has witnessed a net growth of 19% of ES in the past two decades, with a total ES increase of 2.00E+23 sej from 2000 to 2020 (see Table S4). Although the increase is shared across the whole China, most notably in Qinghai-Tibet Plateau, Greater Khingan Mountains, Lesser Khingan Mountains, Changbai Mountains, Taihang Mountains, and Tian Shan (Figure 1A), the decrease is mainly concentrated in the east of Greater Khingan Mountains, Qinghai-Tibet Plateau, Chongqing and Shandong provinces, Dongting Lake, Poyang Lake and the reach of Yangtse River in Shanghai (Figure 1A).

Figure 1B presents the change in ES of each province. The results indicate that in aggregate, five provinces, i.e., Shanghai, Fujian, Henan, Hainan and Shandong, out of 31 provinces in China exhibited a decrease of ES during 2000–2020, with the decrease rate of −15%, −8%, −6%, −4% and −1% respectively (Figure 1B and Table S5). Comparison of Figure 1A with 1b shows that although some specific ES exhibited a decrease in selected provinces (e.g., Tibet), the total ES of the provinces still increase. The opposite is true in Henan and Fujian, where the percent of change in individual ES in Figure 1A suggests an increase, whereas when aggregated at provincial scale, an overall decrease is observed. This seeming contradiction between these two maps is related to the different land use areas involved in the analysis as well as the value of each ecosystem service per unit area.

### Land use change is the dominant driver of the increase in China’s ES

Three drivers are quantified in relation to China’s ES variations: natural drivers R (such as insolation, evapotranspiration (ET), precipitation, NPP, elevation, etc.); human driver S (i.e., land use change listed in Table S1); cognition degree driver *τ* (i.e., the significance degree of human attention to ES improvement) (see STAR Methods). Among them, the growth of ecosystem areas, i.e., appropriate and green-oriented land use, contributes most (55%) to the increase in China’s ES, followed by the improvements of natural drivers (37%), and cognition degree driver (8%) and error (1%) (see Table S6).

**Figure 2:**
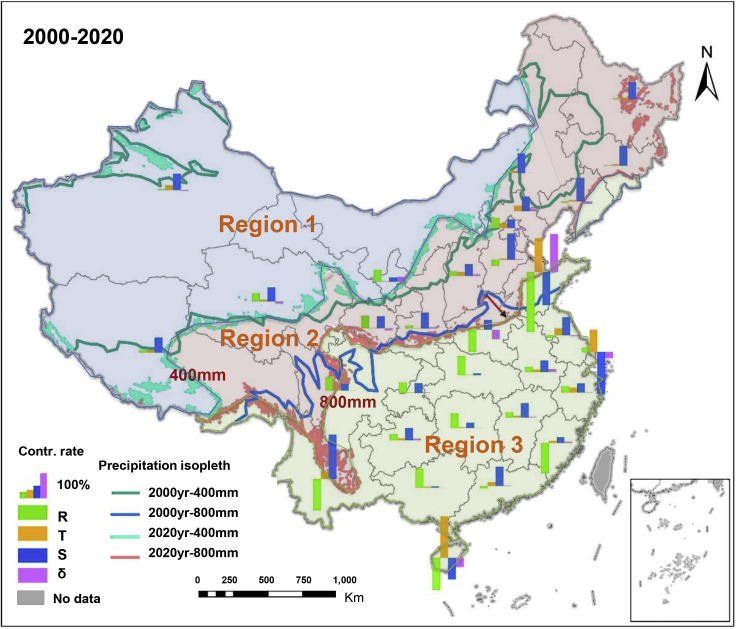
The contribution rate of R, τ, S and δ to the changes in China’s ecosystem services Contr. Rate: Contribution rate; R: Natural drivers (such as precipitation, evapotranspiration, etc.); τ: Cognition degree driver (The significance degree of human attention to ecosystem services improvement); S: Human driver (land use change); δ: Errors.

First, considering 26 provinces which are recorded an ES increase, the main driver for the 18 provinces is the increase in ecosystem areas, represented by the blue bar in Figure 2 (the largest contribution rate), ranging from 44% (Zhejiang Province) to 208% (Yunnan) (Figure 2 and Table S6). This means that 77% of the ES increase in China result from an increase of ecosystems area (see Table S6). In addition, 11 of these 18 provinces are located in the area north over 800 mm precipitation isoline (Figure 2). This indicates that, in areas with relatively poor natural conditions, human efforts to increase the ecosystem areas are effective in improving China’s ES. Among these 18 provinces, Yunnan, Guangdong, Jiangsu, Jiangxi, Guizhou, Anhui and Zhejiang (south of 800 mm precipitation isoline) have more favorable precipitation and heat combinations than the other 11 provinces. Nonetheless, the increase in ecosystem areas is still the dominant driver contributing to the ES improvement in these 7 provinces (Figure 2 and Table S6). These results evidence the relevance of human actions in improving ecosystems and their services in areas with better natural conditions.

Second, the ES improvement in 8 provinces is mainly related to the improvement of natural conditions (Figure 2 and Table S6). Although 18% of China’s areas (the 8 provinces) improve their ES mainly through the improvement of natural conditions (Table S6), afforestation projects still cover each province in mainland China (see Figure S1), suggesting human’s efforts to improve ES in the areas with both favorable and unfavorable natural conditions. These natural conditions include ET, precipitation, elevation, NPP, and biomass carbon density of ecosystem (Table 1). Table 1 shows that in these 8 provinces, the improved natural conditions is related to the increase in ET, with an increase rate of 84.03%–100% (Table 1). ET estimates based on remote sensing further corroborate these findings, i.e., a significant increase in global terrestrial ET in the last four decades, consequently, enhancing water exchange between terrestrial ecosystem and atmosphere (Zeng et al., 2018). Please note that although Earth’s greening and climate change explain 52% and 46% of the increase in global ET respectively (Zeng et al., 2018), the increase in ET is still a natural driver influenced by these factors.

**Table 1:** The improvement or deterioration of specific natural conditions in the provinces dominated by natural drivers

State of natural drivers	Provinces	Natural drivers				
		ET	P	El	NPP	BCD
Improvement	Beijing	100.00%				
	Ningxia	100.00%				
	Hubei	97.30%	2.70%	0.003%		
	Gansu	97.07%	3.27%	−0.05%		−0.29%
	Chongqing	94.17%	5.54%	0.29%		
	Guangxi	92.91%	7.09%			
	Hunan	88.97%	11.03%			
	Sichuan	84.03%	15.74%	0.22%	0.00%	
Deterioration	Shandong	100%				
	Henan	96.40%	3.60%		−0.01%	
	Fujian	85.93%	14.07%			

ET, Evapotranspiration; P, Precipitation; El, Elevation; NPP, Net primary productivity; BCD, Biomass carbon density.

Third, the increase in the degree of human attention has not been the dominant driver of provincial ES improvement. The higher rate of this factor mainly concentrated in relatively developed areas, such as Beijing, Tianjin, Jiangsu, Zhejiang, etc. (Figure 2 and Table S6). This result might depend on the better economic conditions of these provinces, also reflects higher amounts of medical expenses per capita (see Table S7). Such higher total medical expenses reflect greater attention to health care, thereby the greater human attention to ecosystem service improvement.

Concerning the five provinces with declining ES, the decrease in ecosystem areas is the main driver of the ES declining in Shanghai, with a contribution rate of 163% (Figure 2 and Table S6). The degradation of natural conditions is the key drivers causing the decrease of ES in Shandong, Hainan, Fujian, and Henan, with the contribution rates of 280%, 149%, 141%, and 105% respectively (Figure 2 and Table S6). Specifically, ET and precipitation contribute by 100% and 0%, 96.40% and 3.60%, 85.93% and 14.07% to the degradation of natural conditions in Shandong, Henan, and Fujian (Table 1). This is consistent with the observations of year 2020 being the “dry year” of Hainan, the southward movement of 800 mm precipitation contour from 2000 to 2020 in Henan Province and the decrease in precipitation in 2020 in Fujian because of El Nino (Figure 2 and Table S8).

### Region between 400 and 800mm precipitation isohyet is the key area with ES declining

According to natural conditions, we classify the 31 Chinese provinces into three regions (Table 2): (1) Region north over 400mm precipitation isoline (region 1), (2) region between 400 and 800 mm precipitation isoline (region 2), and (3) region south of 800 mm precipitation isoline (region 3). Table 2 shows that, in aggregate, the ratio of natural and human drivers in the area north over 400 mm precipitation line is about 2:7; whereas, the ratio of natural and human drivers in the area south of 800mm precipitation line shows a reversed feature, around 6:3. The contribution of natural drivers in the region between 400 and 800 mm precipitation lines is negative, down to −1%; whereas, the contribution of human drivers is 91%, indicating that human drivers compensate to a certain extent for the decline in ES brought by deteriorating natural conditions in this region. Specifically, the human driver S, dominates the ES improvement in the area north over 800 mm precipitation isoline (regions 1 and 2), with the contribution rates of 71% in the area north over 400 mm precipitation isoline (region 1) and 91% in the area between 400 and 800 mm precipitation isoline (region 2), respectively. Instead, the natural drivers respectively provide a positive and a negative contribution in these two regions, with the rates of 20% and −1%. This is because of the decrease in ET in the area between 400 and 800 mm precipitation line, as well as the southward movement of 800 mm precipitation isoline from year 2000 to 2020. This phenomenon is especially strong in Henan Province (Figure 2). For the area south of 800 mm precipitation isoline, the contribution ratio of nature drivers R and human driver S to ES improvement is about 6:3, highlighting the obvious significance of natural conditions contributing much more to the ES improvement in this region.

**Table 2:** The contribution of different drivers and ecosystems to ES changes from 2000 to 2020 in China’s three regions

Items	Drivers	Regions		
		North of 400 mm	400–800 mm	South of 800 mm
Contribution	R	1.26E+22	−2.94E+20	6.12E+22
	τ	6.63E+21	2.74E+21	6.68E+21
	S	4.59E+22	2.66E+22	3.65E+22
	δ	−8.16E+20	6.41E+19	1.93E+21
	Subtotal	6.43E+22	2.91E+22	1.06E+23
	Ratio	32%	15%	53%
	Subtotal·m^−2^(1)	1.21E+10	1.71E+10	4.07E+10
	Subtotal·m^−2^ (2)	5.00E+10	4.31E+10	1.07E+11
Contribution rate	R	20%	−1%	58%
	τ	10%	9%	6%
	S	71%	91%	34%
	δ	−1%	0%	2%
Contribution rate	Forest	38%	120%	30%
	Shrub	13%	−4%	14%
	HCG	−3%	4%	3%
	MCG	10%	1%	4%
	LCG	5%	3%	1%
	Wetland	2%	1%	0%
	Lake	8%	0%	0%
	R/P	1%	1%	0%
	River	26%	−28%	48%

R: Natural drivers (such as precipitation, evapotranspiration, etc.); τ: Cognition degree driver (The significance degree of human attention to ecosystem services improvement); S: Human driver (land use change); δ: Errors; Ratio is the ratio of the change in ecosystem services in each region to the total change in China’s ecosystem services; Subtotal·m^−2^ (1) is the ratio of the changes in ecosystem services in each region to the total area of each region; Subtotal·m^−2^ (2) is the ratio of the changes in ecosystem services in each region to the total ecosystems area of each region; HCG, MCG, LCG: High, moderate and low coverage grassland respectively; R/P: Reservoir or pond.

Previous studies suggested that the current afforestation actions need to be improved in the region north over 400 mm precipitation isoline, because of their adverse environmental conditions (Bond, 2016; Chen et al., 2015; Liu et al., 2013; Wang et al., 2019). Promising alternative methods include either a natural restoration or a quasi-natural afforestation (Wang et al., 2019; Yang et al., 2016). However, few studies focus on the improvement of ecological conservation practices in the transitional area between 400 and 800 mm precipitation isohyet. Our results suggest that more attention should be paid on this region. Specifically, this study found that it is the key area with observed ES decrease in the past two decades. In particular, both ET and precipitation in Henan province decreased significantly in the past two decades (Table 1, Figure 2 and Table S8). The grassland and lake area in Shandong province (see Table S9), the shrub ecosystem area in Jilin and Heilongjiang (see Table S4) also decreased dramatically. The reasons for the ES decrease in these regions in the past two decades can be summarized as follows: (1) The southward shift of the precipitation line because of climate change makes this area more sensitive; (2) precipitation and policy factors make the land use patterns (agricultural land, shrubs, etc.) change from ecological land to agricultural land and artificial land greatly in this area; (3) this area mainly includes the Yellow River Basin (YRB), and the decline in ES downstream may also be related to the development of upper and middle reaches.

There are specific reasons, confirmed by data and literatures, that caused the observed trends. First, the reduction in rainfall is caused by climate change. There is increasing evidence indicating that climate change leads to a drier north China (Cao, 2008; Li et al., 2018). Just considering precipitation data and assuming that the precipitation line will continue to move south, taking the 400 mm precipitation line as an example, the provinces along the line, from north to south, are mainly Inner Mongolia, Hebei, Shanxi, Shaanxi, Ningxia, Gansu, Qinghai, and Tibet. According to the results of attribution analysis (Figure 1 and Table S6), among these 8 provinces, only the trends observed for Gansu and Ningxia provinces are dominated by natural drivers. Considering ET rather than precipitation as the key natural driver (Table 1), and the 800 mm precipitation line would move south, thus involving Shandong, Henan, Shaanxi, Sichuan and Yunnan provinces. Among these 5 provinces, Sichuan, Henan and Shandong provinces are dominated by natural drivers, among which ET is the most relevant one (Table 1). Results indicate that when the dominant natural driver is ET a slight southward movement of the isoprecipitation lines (such as 400 mm or 800 mm) has a relatively small effect on the provinces along the isoprecipitation lines. Instead, when the dominant natural driver is shifted to precipitation, the southward movement of the isohyet would cause the decline in ES in the provinces along the isoprecipitation line. The ES improvement in the area north over 400 mm precipitation line and the area between 400 and 800 mm precipitation line are dominated by the increase in ecosystem areas. If the precipitation is reduced or, at least, remains unchanged, the extra re-vegetation would generate more water resource pressure in region 1 and 2 than that of other regions, where changes in ecosystem area is not the key driver of ES dynamics. In this case, vegetation would reduce runoff, in turn, further reducing the amount of available water for human activities. This may generate adverse socioeconomic consequences. This type of pressure would be larger in the area between 400 and 800 mm precipitation line than the area north over the 400 mm precipitation, because of the negative contribution of natural drivers in the former area and the frequent fluctuation of the 800 mm isoprecipitation line, especially in Henan and Shandong. This suggests a tradeoff between the carrying capacity of water resources and the scope and intensity of afforestation projects (Chen et al., 2019; Feng et al., 2016), such as a shelterbelt system with an appropriate proportion of trees, shrubs, and grasslands, and moderate vegetation restoration area based on local resources limits, etc. (Wang et al., 2019).

This study proves that the reduction in grassland and lake ecosystem areas in Shandong Province is mainly because of the conversion of these two types of land use into agricultural land and reservoir pond land (see Tables S10A and S10B). Tables S10A and S10B shows that, for Shandong Province, the area converted into agricultural land accounts for 69% of the net loss of grassland ecosystem area, and the area converted into reservoir or ponds accounts for 83% of the net loss of a lake ecosystem area. Consequently, these results suggest that the existing problem is an exceeding economic activity in this province. Located in the North China Plain, Shandong is a province with large population, agriculture and grain production. Its flat terrain and convenient irrigation conditions are favorable for farming. In the past two decades, Shandong Province has replaced wetlands, lakes, grasslands with farmland, thus turning ecological functions into agriculture products provision. Yet, because of the offset of agriculture ecosystem services and dis-services (Shah et al., 2019), the agriculture ES accounting is not included in this study. The decrease in wetlands is also related to intensive land reclamation and other human activities during 2000–2020. Under such a circumstance, it is necessary for local government to make medium- and long-term plans for land use in face of the potential frequent swing of the 800mm isohyet line and vigorous development of human activities. If the converted farmland needs to maintain its current scale, it is necessary to conduct seasonal overall regulation and control of the use of water resources. The harm is particularly severe when reduction in precipitation occurs during the growing season of crops, which means that seasonal regulation of water resources in these areas is extremely significant (Piao et al., 2010). Moreover, modern ecological water-saving agriculture should be promoted, together with an optimization of scale and layout of agricultural land. If it is necessary to implement projects such as returning farmland to forests or shrubs or grasslands, reasonable plans on how to transfer Grain to Green are required. The conversion of land use types from lakes to reservoirs and ponds is actually a conversion from natural water bodies to artificial water bodies. Therefore, it is a big challenge for policy-makers to recover the artificial aquatic area to natural water bodies since this practice implies a large loss of socioeconomic values (Ma et al., 2019; Vaissière et al., 2017). The decrease in shrub ES is attributed to the reduction in shrub ecosystem area (human driver S), with a decrease rate of 17% and 68% in Jilin and Heilongjiang respectively (see Table S11). The transformation of shrub ecosystems to forest ecosystems mainly results in the decrease of shrub area in Jilin and Heilongjiang provinces, with the contribution rate of 104% and 48% respectively (see Tables S10C and S10D)), which is consistent with the findings that the structure and composition of woodland ecosystems in Northeast China would experience large changes due to climate change (Li et al., 2018). Although the decline in the area of shrub ecosystems in Jilin and Heilongjiang Provinces leads to the decline in their shrub ES, the conversion of their shrub ecosystems into forest ecosystems generates an increase in their forest ES. Finally, the total ES in these two provinces shows an increasing trend during 2000–2020. Therefore, the decline in the area of shrub ecosystems do not cause a reduction in their total ES.

The influence between the upper and lower reaches of watershed should not be ignored. For the Yellow River Basin, the decline in ES is mainly concentrated in the middle and lower reaches of YRB. It suggests that the pressure of ecosystem conservation in the middle and lower reaches provinces of the YRB is relatively greater than that in the upstream provinces, which is closely related to the interaction between the upstream and downstream ecosystems. Specifically, the decrease in ES in Shanxi Province is mainly concentrated in wetland and lake ecosystems. Henan Province also needs to focus on the conservation of wetland and lake ecosystems, as these two ES declined by 96% and 97% in the past two decades (see Table S4). Conversely, the area of lakes or reservoir pits in upstream provinces including Sichuan, Gansu, Qinghai and Ningxia increased. While, the opposite trend is observed in the midstream and downstream provinces including Shaanxi, Shanxi and Henan. It may because a large number of intensively constructed reservoirs in the upstream formed a large-scale water storage system, resulting in the shrinkage of wetland, lake and river ecosystems in downstream. In addition, Shandong Province also needs to focus on the conservation and restoration of grassland ecosystems, because the decrease rate of its grassland ES (−29%, −39% and −36%) is significantly larger than that of lake ecosystem (−3%) (see Table S4). The lake ES decreased by 57% in the middle reaches of Shaanxi Province; while, for the upstream provinces, the ES increased in the past two decades with varying increase rates, excepting for Qinghai Province, with a slight decrease (−5%) in wetland ES. Integrally, the decline in ES in provinces and municipalities in the Yellow River Basin is mainly observed for grasslands and aquatic ecosystems. These should be the target for conservation and restoration actions. The increase in ES is mainly concentrated in forest and shrub ecosystems in the Yellow River Basin, resulting from China’s implementing ecological restoration projects, such as SLCP, NFCP and TNSP.

Therefore, compared with the previous studies, which focused on the area north over the 400 mm precipitation line, this study found that the area between 400 and 800 mm precipitation line is more fragile and sensitive, requires more flexible and adaptable policy support.

### Forest ecosystems lead the increase in China’s ES

**Figure 3:**
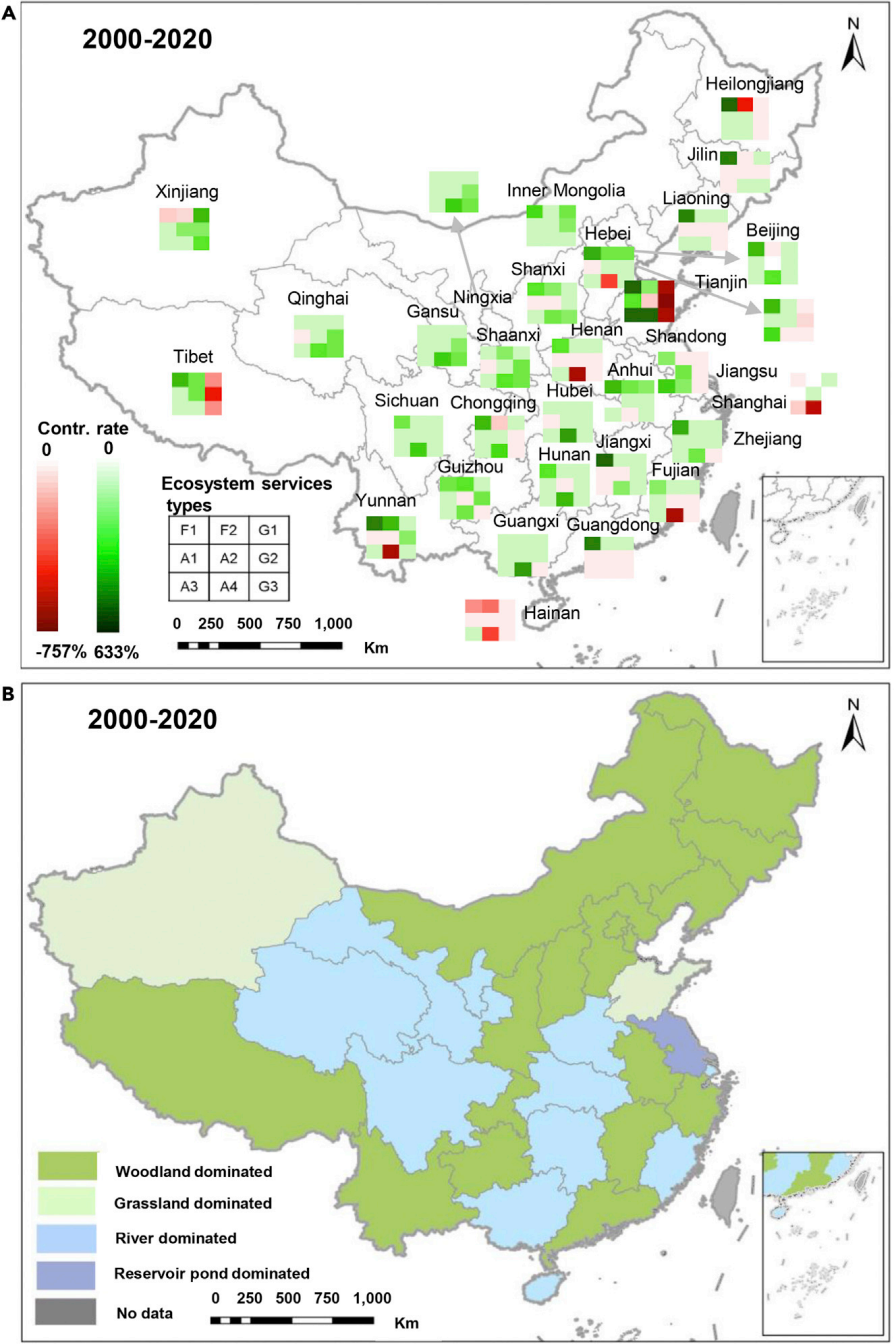
Ecosystems’ contribution to the changes in ecosystem services (A) The percentage of changes in nine ecosystems’ services to the changes of provincial ecosystem services (F1: Forest; F2: Shrub; G1: High coverage grassland; G2: Medium coverage grassland; G3: Low coverage grassland; A1: Wetlands; A2: Lake; A3: Reservoir or pond; A4: River); (B) Diagram of different ecosystems leading contributions to changes in local ecosystem services.

The extent to which ecosystems accounted for the large-scale increase in China’s ES is further investigated. Aggregately, the results show that 48%, 16% and 36% of the increases in China’s ES stem from woodlands, grasslands and aquatic ecosystems. Specifically, 16 provinces are primarily driven by forest ecosystems, contributing 24% (Shaanxi) to 134% (Heilongjiang) to the increase of ES, which are defined as forest-dominated provinces in this study (Figure 3 and Table S12). River ecosystems dominate the increase in ES in 7 provinces, translating into 34% (Qinghai) to 87% (Hubei) in the case of China’s corresponding provinces (Figure 3 and Table S1). The ES improvement in Guizhou, Xinjiang and Jiangsu are attributed to the improvement of their shrub, grasslands and reservoir (or pond) ecosystems respectively (Figure 3 and Table S12). Finally, 3 out of the 5 ES decreased provinces, i.e. Shanghai, Hainan and Shandong mainly derive their results from river ecosystems (Shanghai and Hainan) and grasslands (Shandong) respectively (Figure 3 and Table S12).

These 16 forest-dominated provinces, as well as Guizhou and Xinjiang, are mainly affected by the increase in their forest, shrub and grassland ecosystem areas (see Table S13). Thus, the study on correlation coefficients between the independent variables associated with the changes in woodland and grassland areas and each ecosystem service is further deepened (Table 3). Overall, the results suggest that forestry investment, afforestation area through China’s key forestry projects (see Figure S1) and forestry pest control statistically significantly contribute to the increase in woodland and grassland ES whereas forest fires statistically significantly reduce the ES. Not all independent variables are significantly related to the sub-services of woodland and grassland ecosystems. Just soil retention and microclimate regulation are significantly related and with better significance (p < 0.01) to most independent variables, indicating that these two sub-services are more sensitive and can be predictors of further ES improvements. Specifically, investment and afforestation projects significantly account for the improvement of soil building, groundwater recharge, soil retention, and microclimate regulation. For soil retention and microclimate regulation, the coefficients for the forestry pest control indicate a statistically significantly positive correlation; whereas forestry pest occurrences are significantly negative correlated to these two services. Forest fires are negative and statistically significant (p < 0.05 or p < 0.01) to the increase in most ES. All of these parameters highlight the significance of human actions to improve the quantity and quality of woodlands and grasslands for the increase in China’s ES.

**Table 3:** The correlation coefficient and regression relationship between forestry projects and ecosystem services

Independent Variables	NPP	CS	SB	GR	AP	SR	MR	CR
Forestry investment in 2000	–	–	0.493∗∗	0.374∗	0.514∗∗	0.473∗∗	0.598∗∗	0.525∗∗
Afforestation area completed by key forestry projects in 2000	–	–	0.574∗∗	0.386∗	–	0.558∗∗	0.590∗∗	–
Cumulative afforestation area completed by key forestry projects in 2001 to 2020 ^a^	–	–	0.519∗∗	0.366∗	–	0.575∗∗	0.665∗∗	0.408∗
Area affected by forest fire in 2000	–	–	–	−0.428∗	−0.550∗∗	−0.434∗	−0.359∗	−0.363∗
Differences in area affected by forest fire in 2000–2020^b^	–	–	–	–	−0.440∗	–	–	–
Cumulative area affected by forest fire in 2001–2020^b^	–	–	–	−0.449∗	−0.529∗∗	−0.425∗	−0.372∗	–
Differences in affected area of forestry pest in 2000–2020^a^	–	–	–	0.356∗	–	0.398∗	–	–
Cumulative forestry pest occurrence area 2001–2020^a^	–	–	–	–	–	−0.408∗	−0.465∗∗	–
Forest pest control area in 2000	–	–	–	0.356∗	–	–	–	–
Differences in forestry pest control in 2000–2020^a^	–	–	–	–	–	0.406∗	0.483∗∗	0.378∗
Cumulative forest pest control area in 2001–2020^a^	–	–	0.406∗	–	–	0.456∗∗	0.460∗∗	–
SN	31	31	31	31	31	31	31	31

∗p < 0.05; ∗∗p < 0.01; ∗∗∗p < 0.001; CS, Carbon sequestration; SB, Soil building; GR, Groundwater recharge; AP, Air purification; SR, Soil retention; MR, Microclimate regulation; CR, Climate regulation; SN, Sample size; the units of investment and area are 10,000 yuan and hectare respectively; a, b mean due to the lack of in 2020, the corresponding data in 2019 and 2017 are used here.

## Discussions

### Policy implications

The regions north over 400 mm, between 400 and 800 mm, south of 800 mm precipitation isoline respectively contribute 32%, 15%, and 53% to the increase of China’s ES in the past two decades respectively (Table 2). Although the contribution of the human driver in the area north over 400 mm contour line is around 1.72 times larger than that of in the area between 400 and 800 mm, with values of 4.59E+22 and 2.66E+22 sej/yr respectively, the contribution of human driver per unit area of the region 1 is just 1.16 times than in region 2, with values of 5.00E+10 and 4.31E+10 sej·m^−2^·yr^−1^ respectively. These imply that to achieve the same quality of ES improvement region 1 needs 57% more human investments than region 2. In the area south of 800 mm precipitation isopleth, the contribution per unit area of human driver is the largest (1.07E+11 sej·m^−2^·yr^−1^), suggesting that the existing ecological conservation policies in this area are effective and can be maintained.

### Area north over 400 mm precipitation isohyet needs policies adjustment because local actual NPP is already above water resource limits

The area north over 400 mm precipitation isohyet contributes 32% to the increase in China’s ES. Around 71% of this amount derives from the increase in ecosystem area, which is related to the implementation of the TNSDP since 1978. Some studies found that the greening of desert areas in northwest China could strain water resources (Feng et al., 2016; Fu et al., 2017; Zastrow, 2019). Therefore, the sustainability of water resource use should be considered in relation to the continuous expansion of this ecosystem area north over the 400 mm precipitation line. A study, taking the Loess Plateau as an example, defined the threshold of the regional vegetation capacity by subtracting human water demand from the precipitation, as the permissible NPP for the coupled anthropogenic–biological system (Feng et al., 2016). The same method is applied in this study to calculate the allowable NPP threshold for vegetation restoration in the area north over 400 mm precipitation isoline (Table 4). The results show that the actual NPP of Inner Mongolia, Gansu and Qinghai provinces in the area north over the 400 mm precipitation line are already above the NPP threshold allowed by the local water resource limits, whereas the National Forestry and Grass Administration stated that after the completion of the fifth phase (2011–2020) of TNSDP, a total of approximate 30 million hectares of afforestation and preservation area should be reached. The entire TNSDP is planned to afforest 35.6 million ha by 2050. That means around extra 5.6 million ha will be afforested in this area in the third stage (2021–2050) of TNSDP. More afforestation implies more water demand. Considering that the vegetation restoration in this area has already exceeded the local water resource limits and may cause potential unsustainable problems, such as shortage of human water demand, the local ecological restoration policies need to be adjusted according to local water resources capacity to keep a balance between ecological and socioeconomic water demand (Feng et al., 2016; Han et al., 2021). Policies should be adopted from a holistic perspective, not only including strategies to improve water utilization efficiency in local socio-economic activities but also including the adjustment and optimization of ecological restoration programs. Specifically, adjusting the intensity and extend of ecological programs, i.e., re-vegetation area and vegetation combination (Yang et al., 2022), especially including a better selecting of vegetation species based on local natural characteristics, such as selecting native species because native species is being recognized as an effective way to restore ecosystem functions and services, and to increase biodiversity in degraded areas globally (Chazdon, 2008; Thomas et al., 2014), selecting drought resistance, smoke and dust resistance, and salt-alkali tolerance species, selecting the combination of forests, shrubs and grasslands, together with an appropriate choice of restoration area based on local permissible NPP threshold. These policy implications are highly recommended to ensure the sustainability of water use in the coupled anthropogenic–biological system.

**Table 4:** The comparison of NPP threshold and actual NPP in the area north of the 400 mm isoprecipitation line in 2020

Areas	NPP threshold	Actual NPP
Inner Mongolia	195.11	233.30
Gansu	240.33	332.42
Qinghai	134.36	135.28
Ningxia	220.01	100.24
Xinjiang	68.01	55.92
Tibet	400.02	147.18

The unit of NPP is g C·m^−2^·yr^−1^; the actual NPP is the average NPP of forest and grasses.

### The area between 400 and 800 mm isoprecipitation isohyet is more fragile and requires more policy support

This area contributes 15% to the increase in China’s ES. Parts of this region are still declining in their ES supply, suffering from comprehensive impacts of natural drivers (such as reduction of precipitations caused by climate change) and human drivers (the decrease in ecosystem area), and calling for flexible policies according to local conditions.

Taking the Henan Province as an example, the first reason for its ES decline is the southward of 800 mm contour line during 2000–2020. The largest proportion of water consumption in Henan Province was farmland irrigation in 2020, accounting for around 52% of the total water consumption in the province (WRDHP, 2021). Therefore, the province should vigorously develop water-saving strategies, speed up the construction of modern farmland irrigation and drainage systems, develop large-scale and high-efficiency water-saving irrigation, and improve farmland irrigation water efficiency (WRDHP, 2021). These policies were already included in the “14th Five-Year Plan” (2021–2025) of Henan Province (PGHP, 2021).

Meanwhile, large amounts of ecological lands (mainly grasslands) were converted into agricultural lands. For example, Shandong Province has formulated policies to protect farmland areas from 2001 to 2020, and achieved its farmland retention target (PGSP, 2001; PGSP 2006; PGSP, 2011; PGSP, 2016). However, according to satellite data, the area of farmland still shows a downward trend from 2000 to 2020 (see Figure S2), indicating that Shandong Province is under increasing pressure to maintain the area of farmland. Therefore, on one hand, Shandong Province needs to improve low- and middle-yield farmland and build high-standard farmland that can guarantee income from droughts and floods. On the other hand, under the necessity and urgency of maintaining farmland area and frequently fluctuant precipitation, Shandong Province needs to develop water-saving agriculture to alleviate the water pressure caused by agricultural irrigation.

The third reason is that the construction of a large number of intensive reservoirs in the upper reaches of the Yellow River Basin will lead to the shrinkage of the river, lake and wetland ecosystems in the lower reaches. Therefore, it is necessary to vigorously promote collaborative management of the YRB. First, it is necessary to establish a cross-administrative ecological compensation system in the Yellow River Basin, and the beneficiary areas should provide necessary compensation to those areas that bear the cost of ecological environment protection and construction and lose development opportunities in the river basin. Second, it is crucial to establish a collaborative information platform for the YRB to dynamically reflect the ecological conditions, environmental quality, hydrology, pollution source lists, watershed shoreline management and operation of the basin, to share and communicate the information among the river basin management departments. Third, it is also important to unify the governance planning and environmental impacts assessment in the YRB. Fourth, it needs to establish a coordinated monitoring system for the entire YRB by high-tech ways such as satellite positioning and the internet of things to promote the construction of online monitoring facilities in the form of a multi-element, multi-media dynamic process, toward a full coverage, high-precision, and rapid response three-dimensional monitoring network. These coordinated management strategies were supposed to be implemented at least before 2015 because the construction of reservoirs in the upper reaches has led to the shrinkage of aquatic ecosystems in the lower reaches in 2000–2015 (Yang, 2020), resulting in the decrease in ES in the lower reaches, but they were not carried out until 2019. When the “era of coordinated governance” of the Yellow River Basin was started, it became a national major strategy. It shows that although China’s policy implementations to improve ecological states and environment are relatively lagging on a temporal scale, they are still effective to comprehensively improve ecological status and environment by integrating the entire ecosystem perspective and the specific local conditions.

On the other hand, a large number of natural aquatic ecosystems (i.e., lakes) in the Shandong Province have been transformed into artificial aquatic ecosystems (i.e., reservoirs or ponds). Although the total area of aquatic ecosystems in the Shandong Province shows an increasing trend from 2000 to 2020 (see Table S9), the area of the natural aquatic ecosystem (i.e., lake ecosystem) shows a decreasing trend from 2000 to 2020 (−13%) (see Table S9), which may result in the homogeneity of ecosystem functions, consequently opposite to maintaining the functions of the aquatic ecosystems and to ensuring the sustainable use of water bodies in the long run. Previous studies have shown that tidal flat reclamation, fishery or salt industry development, construction of water conservancy facilities, oil field development, and so on, are the main reasons for the shrinkage of natural aquatic ecosystems and the increase in artificial aquatic ecosystems in the Shandong Province (Gong, 2015). Therefore, Shandong Province needs to properly handle the relationship between agricultural development and aquatic ecosystems conservation, to balance the environmental pressure brought by regional development and population growth, and to not blindly reclaim or transform aquatic ecosystems in pursuit of economic benefits. However, the area of lake ecosystem decreased by only −0.1% from 2015 to 2020 (see Table S9), indicating the effects of the strategies on improving aquatic ecosystems during the “13th Five-Year Plan” period (2016–2020) in Shandong (PGSP, 2016). Furthermore, it needs to strengthen the conservation of natural aquatic ecosystems. Specifically, it requires a continuous curb of the unreasonable development of aquatic ecosystems (such as converting natural water bodies to artificial aquatic ecosystems) as well as effective actions to restore aquatic ecosystems. It needs not only to ensure the area of aquatic ecosystems, but also pay attention to their quality to ensure their functional stability and biodiversity (Gong, 2015).

### The policies are effective in the area south of 800 mm isoprecipitation line and can be maintained

Region 3 contributes 53% to the increase in China’s ES. Natural and human drivers contribute 58% and 34% respectively to this proportion (Table 2), suggesting that less than 40% of human drivers in this area contributed to around 53% of China’s ES improvement in the past two decades. Therefore, the existing policies in this region can be maintained to achieve the trend of further improvement of China’s ES. It should be noted that although the Yunnan-Guizhou, Sichuan, and Chongqing regions belong to the area south of 800 mm precipitation isoline, runoff geopotential energy was relatively higher than the other renewable resources (such as solar energy, etc.). This may be related to the relatively larger altitude, elevation difference and precipitation in the Yunnan-Guizhou Plateau and Hengduan Mountain areas (Yang, 2015). Because of the mountainous terrain, complex landforms and diversified surface vegetation in these areas, it is necessary to combine the actual local conditions with ecological restoration solutions. On-site inspections could be particularly significant. Taking Guizhou Province as an example, land with a slope of ≥25° accounting for 32.27% of the total provincial land area (Wu, 2019), it should be forbidden to reclaim cultivated land on the slopes and return cultivated land to forests or grasslands for water and soil retention. The arable land between 6° and 25° accounts for 63.44% of the total arable land in this province (Wu, 2019), where irrigation and drainage projects should be designed to improve the utilization efficiency of water resources, and the arrangement of arable land parcels should be mostly parallel to the contour lines to improve the utilization efficiency of solar energy resources.

In summary, the ecological protection policies for the area north over the 400 mm precipitation line have improved its ES, yet the policies need further adjustment to adopt to the local water resource limits to ensure the sustainability of water resources utilization and ecological restoration. There has been a decline in ES in some areas between the 400 and 800 mm precipitation line because of the comprehensive impact of climate change, land use change, policy neglect, and so on. This area is more fragile and requires more flexible policy support. The area south of the 800 mm precipitation line contributes more than half of China’s ES improvement with less than one-third of China’s area. It can consider maintaining the current policies.

### Limitations of the study

Because of data availability, this study just identified and assessed the contribution of drivers to China’s ES changes in the past two decades, yet ES dynamics is a product of long-term ecological process and if the contribution of drivers of global ES changes can be assessed, it would be better to provide references for other cases. Therefore, if data is available, further study can expand the spatial-temporal scale to better serve ecosystem management and conservation. In the calculation process of this study, a large number of data are required, such as land use data, NPP, ET, solar radiation, precipitation, and so on, but the data accuracy is inconsistent. To unify data resolution, we use the resample tool in ArcGIS software deal with the raster data, which may cause deviations in the results. Future studies require more precise data to improve the accuracy of the results.

### Conclusions

ES in 89% of China’s ecosystem areas increased from 2000 to 2020, with a net growth ratio of 19%. Human drivers contribute most (55%) to the increase in China’s ES, followed by the improvement of natural drivers (37%), and cognition degree driver (8%) and error (1%). The ratio of natural drivers to human drivers in the area north over 400 mm precipitation isopleth (region 1) is 2:7, whereas the ratio in the region south of 800 mm precipitation contour isoline (region 3) is 6:3. The contribution rate of natural drivers to the ES improvement in the area between 400-800 mm precipitation isopleth (region 2) is negative (−1%), whereas the contribution of human drivers is 91%. The ecological restoration polices in region 1 is effective, yet it needs to adjust because local NPP has already exceeded the water resource limits. Region 2 is more fragile and requires more policy support. Region 3 applies less than 40% of human efforts contributing around 53% to China’s ES improvement, therefore, the policies there can be maintained. On the other hand, there still exist challenges such as lack of the integration of large-scale, long-term and systematic ground observation network with high-resolution remote sensing satellite observations. This combination would improve the accuracy of assessment and confirm the improvement of China’s ES concluded in this study and further facilitate the improvement of current ecological policies. Finally, the proposed attribution analysis method, which is proved to be effective in identifying and assessing the specific contribution of drivers of ES variations at multiple scales, could be applied to other target cases for implementing more refined ecological restoration policies adapting to local conditions.

## STAR★Methods

### Key resources table

**Table:** 

RESOURCE	SOURCE	DENTIFIER
**Deposited Data**		

Land use and land cover (LULC) remote sensing data	Data Center for Resources and Environmental Science, Chinese Academy of Sciences (RESDC)	https://www.resdc.cn/

solar radiation	China Meteorological Administration	https://wenku.baidu.com/view/4479d2c381eb6294dd88d0d233d4b14e85243e2a.html
Precipitation	Statistical Yearbooks of China’s provinces, 2001–2016; China’s Water Resources Bulletin in 2020 (MWRPRC, 2020)	https://data.cnki.net/Yearbook/; http://www.mwr.gov.cn/sj/tjgb/szygb/202107/P020210909535630794515.pdf.
DEM	Data Center for Resources and Environmental Science, Chinese Academy of Sciences (RESDC)	http://www.resdc.cn/data.aspx?DATAID=123
NPP	Data Center for Resources and Environmental Science, Chinese Academy of Sciences (RESDC)	https://www.resdc.cn/data.aspx?DATAID=204
Evapotranspiration	CGIAR Consortium for Spatial Information (CGIAR-CSI)	https://figshare.com/articles/dataset/Global_High-Resolution_Soil-Water_Balance/7707605/3?file=14342702
NDVI	Xu (2018); Data Center for Resources and Environmental Science, Chinese Academy of Sciences (RESDC)	https://www.resdc.cn/data.aspx?DATAID=343

Forestry investment	NFGA (2020); China Forestry and Grassland Statistical Yearbook	https://data.cnki.net/yearbook/Single/N2021060073
Afforestation area completed by key forestry projects	NFGA (2020); China Forestry and Grassland Statistical Yearbook	https://data.cnki.net/yearbook/Single/N2021060073
Area affected by forest fire	NFGA (2020); China Forestry and Grassland Statistical Yearbook	https://data.cnki.net/yearbook/Single/N2021060073
Area affected of forestry pest	NFGA (2020); China Forestry and Grassland Statistical Yearbook	https://data.cnki.net/yearbook/Single/N2021060073
Forest pest control area	NFGA (2020); China Forestry and Grassland Statistical Yearbook	https://data.cnki.net/yearbook/Single/N2021060073

Note: The raster data, i.e., DEM, NPP, ET and NDVI, are resampled using software ArcGIS to gain the same resolution as LULC data, which is 30m × 30m in 2020. For provincial data, all ecosystems are assumed to have the same solar radiation and precipitation in one province because of the lack of raster data.)

### Resource availability

#### Lead contact

Further information and requests for resources and reagents should be directed to and will be fulfilled by the lead contact: Gengyuan Liu (liugengyuan@bnu.edu.cn).

#### Materials availability

This study did not generate new unique materials.

### Method details

#### Emergy-based accounting method of ecosystem services

The Emergy-based ES accounting method includes four parts: Ecosystem classifications (see Table S1), ES classifications (see Table S2), ES accounting techniques, and the summation principles of total ES. Nine types of ecosystems are investigated in this study including: woodlands (including forests and shrubs), grassland (including high, moderate and low coverage grassland), and aquatic ecosystems (including wetlands, rivers, lakes, reservoir or ponds). Eleven ES are computed as follows: NPP, carbon sequestration, soil building, groundwater recharge, air purification, water purification, materials transport, soil retention, hydropower generation (nature’s contribution), microclimate regulation and climate regulation (see Table S2). Not all 11 ES are corresponding to each ecosystem because some ecosystems do not have such services (e.g., forest systems do not have material transport service). Each ES accounting techniques and the total ES summation principles are detailed as follows.

#### Ecosystem areas correction based on vegetation fraction

The same vegetation area may have various vegetation growth and coverage conditions indicating that it needs to correct the land use date from satellite using vegetation coverage indicator. Vegetation fraction (VF), the percentage of the vertical projection of vegetation (including leaves, stems, and branches) on the ground to the total area of the statistical area, is applied to capture the vegetation feature and revise the original land use date in this study. According to Montandon and Small (2008), VF is calculated based on normalized difference vegetation index (NDVI) as follows:

(Equation 1) $${\text{VF}}_{i}=\frac{NDVI_{i}-NDVI_{min}}{NDVI_{max}-NDVI_{min}}$$

(Equation 2) $$S_{i}^{\prime }=S_{i}\cdot VF_{i}$$

where $VF_{i}$ and $NDVI_{i}$ mean the VF and NDVI of the ecosystem *i* respectively; $NDVI_{min}$ and $NDVI_{max}$ are the minimum and maximum NDVI of ecosystem *i* respectively; $S_{i}$ is the area of ecosystem *i* from remote sensing data (m^2^); $S_{i}^{\prime }$ is the area of ecosystem *i* modified by VF and then applied to calculated ES in this study(m^2^).

#### Ecosystem services accounting techniques

According to Table S2, some ES are only linked to specific ecosystems, and a detailed description of the calculation procedure is given below. Most services are instead considered as provided by all or many among the ecosystems considered. The reasons why these techniques are applied to calculate ESs are detailed in (Yang et al., 2018, 2019a, 2019b, 2020).

##### Net primary productivity (NPP)

NPP refers to the photosynthetic gain of plants per unit area, minus respiratory costs (Jmo et al., 2002). Photosynthesis is driven by local renewable resources. Therefore, NPP is calculated as:

(Equation 3) $$Em_{NPP}=\sum_{i=1}^{n}\left(MAX\left(R_{i}\right)\;\right)$$

where $Em_{NPP}$ represents the emergy needed by NPP in a given area (sej/yr); $\text{MAX}\left(R_{i}\right)$ is the maximum value among the renewable resources in ecosystem i (sej/yr). According to Brown and Ulgiati (2016), it can be calculated as follows and the detailed accounting formulas of each renewable resources in Equation (4) can also be found in Brown and Ulgiati (2016).

(Equation 4) MAX(R) = MAX [∑(solar energy, tidal energy, thermal energy), wave energy, wind energy, rain (chemical potential energy), runoff(geopotential energy), runoff (chemical potential)].

##### Carbon sequestration

Terrestrial ecosystems have functioned as significant carbon sinks, accounting for 20–30% of the total anthropogenic carbon dioxide (CO_2_) emissions to the atmosphere (Tang et al., 2018). Vegetation sequestrate CO_2_ through photosynthesis, the same process of net primary production. Therefore, the unit emergy value (UEV) of carbon sequestration can be calculated by this process.

(Equation 5) $$Em_{CS}=\sum \left(\frac{C_{i}}{T_{i}}\cdot S_{i}^{\prime }\cdot UEV_{csi}\right)$$

(Equation 6) $$UEV_{\text{cs}i}=\frac{\left(Em_{NPPi}\right)/S_{i}^{\prime }}{NPP_{i}}$$

where $Em_{CS}$ indicates the emergy used to sequestrate carbon in ecosystems (sej·yr^−1^); $C_{i}$ refers to the carbon sequestrated in ecosystem *i* (g C·m^−2^); $T_{i}$ is the average turnover time of carbon pool in ecosystem *i* (yr); $S_{i}^{\prime }$ represents the area of ecosystem *i* after correction (m^2^);$\;UEV_{\text{cs}i}$ means the unit emergy value (UEV) of carbon sequestrated in ecosystem *i* (sej·g^−1^); $Em_{NPPi}$ is the renewable emergy driving NPP of ecosystem *i* (sej·yr^−1^), which is $Em_{NPPi}$ in Equation (3); $NPP_{i}$ is the NPP of ecosystem *i* (g C·m^−2^·yr^−1^).

##### Soil building

Soil is built through soil organic matter and mineral building. Soil organic matter stems from vegetation litter (a part of biomass). Minerals are mainly formed by parental material through weathering (Campbell, 2012). This service is for forest, shrub and grassland ecosystems.

###### Soil organic matter building

Soil organic matter (SOM) is formed through partial decomposition and transformation of plant inputs by soil organisms (Cotrufo et al., 2015), which means SOM is part of the biomass. Therefore, the ratio of vegetation litter to biomass is applied to evaluate the emergy needed to build SOM.

(Equation 7) $$Em_{\text{OM}}=\sum_{i=1}^{n}\left(Em_{\text{rei}}\cdot k_{1i}\cdot k_{2}\right)=\sum_{i=1}^{n}\left(Em_{NPPi}\cdot k_{1i}\cdot k_{2}\right)$$

where $Em_{\text{OM}}$ indicates the emergy applied to build soil organic matter (sej·yr^−1^); $Em_{\text{rei}}$ is the renewable emergy of ecosystem *i* (sej·yr^−1^), equal to $Em_{NPP}$ in Equation (3); ${\text{k}}_{1i}$ means the ratio of the plant litter to the biomass of ecosystem *i* (g·g^−1^, %), and because of the lack of data, the amount of plant litter of forest ecosystems in various areas in this study applies China’s average amount of plant litter of forest ecosystems, and the biomass of forest ecosystems in different areas is evaluated based on their local biomass carbon intensity (Sun et al., 2015); as to shrub and grassland ecosystems, their indicators, i.e., ${\text{k}}_{1i}$, have specific measurements; ${\text{k}}_{2}$ represents the carbon amount in detritus (g·g^−1^, %).

###### Soil minerals building

Parent rocks are sources of soil minerals through weathering driven by the interaction of geologic processes and climatic factors (Campbell, 2012). Geologic processes can drive various soil minerals formation simultaneously. Therefore, the maximum value of emergy required to form different minerals is taken as the final soil mineral building service and calculated as:

(Equation 8) $$Em_{\text{Min}}=Max((\left(P_{mij}\cdot BD_{j}\cdot D_{j}\cdot S_{j}^{\prime }\cdot R\cdot 10000\right)/T_{i})\cdot UEV_{mi})$$

where $Em_{\text{Min}}$ is the emergy used to build soil minerals (sej·yr^−1^); $P_{mi}$ is the proportion of *i*-th mineral to total soil mineral of ecosystem *j* (%); $BD_{j}$ is the soil bulk density of *j*-th ecosystem (g·cm^−3^); $D_{j}$ represents the soil depth of the *j*-th ecosystem (cm);$\;S_{j}^{\prime }$ is the area of the *j*-th ecosystem after correction (m^2^); R indicates the percentage of soil mineral to total soil mass (%), which is 95% in this study (Liu, 2009); 10000 is the conversion factor from m^2^ to cm^2^; $T_{i}$ is the turnover time of mineral *i* (yr), estimated as 1000 years because of the lack of data; $UEV_{mi}$ indicates the UEV of mineral *i* (sej·g^−1^).

Soil building service is quantified as:

(Equation 9) $$Em_{SB}=Em_{\text{OM}}+Em_{\text{Min}}$$

##### Sediment building

Sediment organic matter is a source of food and energy for aquatic organisms, as well as a source of “recycle nutrients” for waters productivity (Froelich et al., 1979). Meanwhile, its nutritional balance plays a significant role in material flow through ecosystems (Meyers and Teranes, 2001; Westrich and Förstner, 2007).

Sediment building service in this study refers to the organic matter building in sediments in aquatic ecosystems without eutrophication, whereas this service is excluded from the total service when an aquatic ecosystem is eutrophic. The particulate detritus of vegetation is the primary source of lake organic sediments (Lerman et al., 1995). Nearly all organic matter originates from plants; less than 10% come from animals (Meyers and Ishiwatari, 1995). The calculation method of sediment building can be written as follows:

(Equation 10) $$Em_{SBa}=\sum \left(OM_{ai}\cdot k_{1}\cdot k_{2i}\cdot k_{3}\cdot S_{i}^{\prime }\cdot UEV_{omi}\right)$$

(Equation 11) $$OM_{ai}=k_{4}\cdot NPP_{i}$$

where $Em_{SBa}$ is the emergy applied to deposit organic matter in aquatic ecosystems (sej·yr^−1^); $OM_{ai}$ is the deposition of organic matter in aquatic ecosystem *i* (g·m^−2^·yr^−1^); $k_{1}$ is the fraction of deposition absorbed by aquatic plants, which is 0.78 (Mitsch and Gosselink, 1994); $k_{2i}$ is the conversion factor from g to kcal in aquatic ecosystem *i*; $k_{3}$ is the conversion factor from kcal to J, which equals to 4186J·kcal^−1^; $S_{i}^{\prime }$ presents the *i-*th aquatic ecosystem’s area after correction (m^2^); $UEV_{omi}$ is the UEV of organic sediment deposition in aquatic ecosystem *i* (sej·J^−1^);$\;k_{4}$ is the ratio of organic sediment deposition to the NPP in aquatic ecosystem *i*, which is 30.37% (Gale and Reddy, 1994); $NPP_{i}$ is the net primary productivity of aquatic ecosystem *i* (g C·ha^−1^·yr^−1^).

##### Groundwater recharge

Groundwater recharge refers to a hydrologic process, through which surface water enters to groundwater (Freeze and Cherry, 1979). It can be esteemed as:

(Equation 12) $$Em_{GR}=\sum_{i=1}^{n}\left(P_{i}\cdot S_{i}^{\prime }\cdot \rho \cdot k_{i}\cdot 1000\cdot UEV_{\text{wi}}\right)$$

where $Em_{GR}$ indicates the emergy applied to recharge groundwater (sej·yr^−1^); $P_{i}$ means the precipitation in ecosystem *i* (m·yr^−1^);$\;S_{i}^{\prime }$ represents the area of *i* after correction (m^2^); ρ means water density (kg·m^−3^); $k_{i}$ refers to precipitation infiltration coefficient of ecosystem *i* (%); 1000 is the conversion factor from kg to g; $UEV_{\text{wi}}$ indicates the UEV of rainfall (sej·g^−1^).

##### Air purification

Disability Adjusted Life Years (DALYs) and Potentially Disappeared Fraction (PDF) of species are applied to measure air purification service. These factors were introduced in the assessment framework of Eco-Indicator 99 investigated by Huijbregts et al. (2017). The meaning of DALYs and PDF and the reason for selecting them as indicators related to ES were given by Goedkoop and Spriensma (2001) and Yang et al. (2018). Air pollutants including SO_2_, fluoride, NO_x_, CO, O_3_, PM_10_ and PM_2.5_ are investigated in this study. Considering the impacts of pollutants on human and ecosystem are variable, the benefits of air purification are given as the cumulative decline in human health losses and ecosystem quality degradation. The calculation formulas are detailed hereafter.

###### Decline in human health losses

(Equation 13) $$Em_{HH}=\sum_{i=1}^{n}\left(M_{ij}\cdot S_{j}^{\prime }\cdot DALY_{i}\right)\cdot \tau_{H}$$

(Equation 14) $$\tau_{H}=\left(I\cdot EmR\right)/Pop$$

where $Em_{HH}$ is the emergy applied to reduce human health decline (sej·yr^−1^); $M_{ij}$ represents the capacity of ecosystem *j* to absorb the *i*th air pollutant (kg·ha^−1^·yr^−1^); $S_{j}^{\prime }$ indicates the area of ecosystem *j* after correction (ha); $DALY_{i}$ refers to the DALY of one individual generated by the *i*th air pollutant (cap·yr·kg^−1^); $\tau_{H}$ is the emergy corresponding to the total health expenditure per capita in the region (sej·cap^−1^); I represents the total cost of medical and health investment in the area (yuan), which is composed of three parts: government, society and individual health expenditures, and reflects the payment level and significance degree of government, society and individual for health care under a certain economic condition. This data stems from 2001 to 2016 China Health and Family Planning Statistical Yearbook. The total health expenditure per capita is the ratio of total health expenditure in a certain year to the average population during the same period.

###### Decline in ecosystem quality degradation

(Equation 15) $$Em_{EQ}=\sum_{i=1}^{n}\left(M_{ij}\cdot PDF_{i}\cdot Em_{spj}\right)=\sum_{i=1}^{n}(M_{ij}\cdot PDF_{i}\cdot MAX\left(R_{j}\right))$$

where $Em_{EQ}$ indicates the emergy needed to decrease the ecosystem quality degradation (sej·yr^−1^); $M_{ij}$ has the same meanings as $M_{ij}$ in Equation (13); $PDF_{i}$ represents the PDF of species brought from air pollutant *i* (PDF·ha·yr·kg^−1^); $Em_{spj}$ means the emergy needed by the species in ecosystem *j* (sej·yr^−1^); $MAX\left(R_{j}\right)$ has the same meaning as $MAX\left(R_{i}\right)$ in Equation (3) with the exception of different subscripts.

Air purification service ($Em_{AP}$) is measured as:

(Equation 16) $$Em_{AP}=Em_{HH}+Em_{EQ}$$

##### Water purification

Aquatic ecosystems have the capacity to remove contaminants from water by a variety of processes (Ostroumov, 2004), including dilution, sedimentation, aeration, absorption, floatation and chemical and biological reactions (González et al., 2014). When the concentration of pollutants exceeds the self-purification ability of water bodies, the capacity will not work (González et al., 2014; Yang et al., 2009). Hence, the self-purification capacity of aquatic ecosystems is selected to evaluate the water purification service. In this study, because of the availability of date, heavy metals in water are selected as water pollutants, including chromium (Cr), nickel (Ni), copper (Cu), manganese (Mn), zinc (Zn), cadmium (Cd), and lead (Pb).

###### Reduction in human health losses

(Equation 17) $$Em_{HH}=\sum \left(\left(M_{ij}\cdot NPP_{j}\cdot S_{j}^{\prime }\cdot DALY_{pi}\cdot \tau_{H}\right)/T_{i}\right)$$

where $Em_{HH}$ means the emergy needed to reduce damages to human health (sej·yr^−1^); $M_{ij}$ presents the ability of the *j*th aquatic ecosystem to remove water pollutant *i* (mg·kg^−1^);$\;NPP_{j}$ is the net primary productivity of aquatic ecosystem *j* (g C·m^−2^·yr^−1^); $S_{j}^{\prime }$ is the *j*th aquatic ecosystem’s area after correction (m^2^); $DALY_{pi}$ is the DALY of one individual resulted from *i*th water pollutant (cap·yr·kg^−1^); $\tau_{H}$ presents the same meaning as Equation (13); $T_{i}$ indicates the *i*th water pollutant’s turnover time (yr).

###### Reduction in ecosystem quality losses

(Equation 18) $$Em_{EQ}=\sum \left(M_{ij}\cdot NPP_{j}\cdot PDF_{pi}\cdot Em_{spj}\right)/T_{i}$$

where $E_{mEQ}$ presents the emergy used to reduce damages to ecosystem quality (sej·yr); $PDF_{pi}$ means the PDF of species caused by the *i*th water pollutant (PDF·m^2^·yr·kg^−1^); $Em_{spj}$ indicates the emergy needed by species in aquatic ecosystem *j* (sej·yr^−1^), which is expressed as local renewable resources (sej·yr^−1^) and can be calculated by Equation (4); $M_{ij}$, $NPP_{j}$ and $T_{i}$ have the same meanings as the ones in Equation (17).

The total water purification value $Em_{WP}$ is the sum of $Em_{HH}$ and $Em_{EQ}$.

##### Soil retention

Generally, on relatively flat grassland and/or forest-covered land, erosion rate ranges from a low level of 0.001–2t ha^−1^·yr^−1^ to a rate of 1–5t ha^−1^·yr^−1^ on mountainous areas with normal vegetation cover (Pimentel and Kounang, 1998). Therefore, this service is for forest, shrub, and grassland ecosystems. This soil retention ability is quantified as:

(Equation 19) $$E_{mSR=}\sum_{i=1}^{n}\left(G_{i}\cdot S_{i}^{\prime }\cdot r_{omi}\cdot 10^{6}\cdot k_{r1}\cdot k_{r2}\cdot UEV_{sl}\right)$$

where $Em_{SR}$ is the emergy needed by soil retention (sej·yr^−1^); $G_{i}$ represents the amount of soil retention because of the cover of ecosystem *i* (t·ha^−1^·yr^−1^); $S_{i}^{\prime }$ indicates the area of ecosystem *i* after correction (ha); $r_{omi}$ is the soil organic matter content in ecosystem *i* (%); $10^{6}$ is the conversion factor from ton to gram (g·t^−1^); $k_{r1}$ is the conversion factor from g to kcal (kcal·g^−1^); $k_{r2}$ is the conversion factor from kcal to J (J·kcal^−1^); $UEV_{sl}$ is the transformity of soil (sej·J^−1^).

##### Microclimate regulation

Ecosystems regulate microclimate through increasing humidity, precipitation and decreasing temperature. Because the energy absorbed during evapotranspiration equals that of increasing humidity and decreasing temperature in ecosystem, the energy required by evapotranspiration can be used to measure the humidity increase and temperature decrease values. The calculation is as follows:

(Equation 20) $$Em_{MR}=\sum_{i=1}^{n}\left(E_{ei}\cdot S_{i}^{\prime }\cdot UEV_{et}\right)$$

where, $Em_{MR}$ represents the emergy applied to regulate microclimate (sej·yr^−1^); $E_{ei}$ is the evapotranspiration in ecosystem *i* (g·m^−2^·yr^−1^); $S_{i}^{\prime }$ is the area of ecosystem *i* after correction (m^2^); $UEV_{et}$ is the UEV of water transpiration (sej·g^−1^).

##### Materials transport

Materials transport refers to the movement of solid particles, generally because of the acting of gravity on the materials and/or the movement of the fluid, in which the materials are contained (Czuba, 2018). Materials transport is significant in providing habitat for fish and other organisms in rivers (Valero et al., 2017). In this study, river ecosystem has this service. Transported materials include nutrients, organic matter and sediments. Driven by geopotential energy, materials transport is assessed as follows:

(Equation 21) $$Em_{MT}=\sum \left(S_{i}^{\prime }\cdot R_{ai}\cdot \rho \cdot k_{r}\cdot h_{i}\cdot g\cdot UEV_{rgeo}\right)$$

where $Em_{MT}$ is the emergy required to transport materials in river ecosystem (sej·yr^−1^); $S_{i}^{\prime }$ presents the *i*th aquatic ecosystem’s area after correction (m^2^); $R_{ai}$ means the rainfall in aquatic ecosystem *i* (m·yr^−1^); ρ presents water density (kg·m^−3^); $k_{r}$ indicates runoff rate, which is 25% (Brown and Ulgiati, 2016); $h_{i}$ is the average elevation of aquatic ecosystem *i* (m); g is the gravity, which is 9.8 m s^−2^; $UEV_{rgeo}$ is the transformity of runoff (geopotential energy) (sej·J^−1^).

##### Hydropower potential (nature’s contribution)

Hydroelectricity is generated in a dam, where the force of falling water is used to turn a turbine that is connected to an electricity generator (Xu et al., 2018a). Hydropower is the most widely exploited form of renewable energy with very few greenhouse gases emissions (Solarin et al., 2019). Hydropower is derived from the combination action of runoff and elevation difference, which are driven by rainfall and mountain building respectively. Therefore, the measurement of hydropower service is:

(Equation 22) $$Em_{HG}=Em_{r}+Em_{mb}$$

(Equation 23) $$Em_{r}=\sum \left(S_{dci}\cdot R_{di}\cdot \rho \cdot UEV_{r}\right)$$

(Equation 24) $$Em_{mb}=\sum \left(S_{dci}\cdot r_{di}\cdot 10^{6}\cdot \rho_{m}\cdot UEV_{m}\right)$$

where $Em_{HG}$ is the emergy required to generate hydropower in river ecosystem (sej·yr^−1^); $Em_{r}$ is the emergy contributed by rainfall to generate hydroelectricity in river ecosystem (sej·yr^−1^); $Em_{mb}$ is the emergy contributed by mountain building to form hydropower in river ecosystem (sej·yr^−1^); $S_{dci}$ presents the catchment area of dam *i* in river ecosystem (m^2^); $R_{di}$ is the rainfall in dam *i* area (m·yr^−1^); ρ presents water density (kg·m^−3^); $UEV_{r}$ indicates the UEV of rain (sej·g^−1^); $r_{di}$ means average deviation rate in dam *i* area in river ecosystem (m·yr^−1^); $10^{6}$ is the conversion factor from m^3^ to cm^3^, which means 1m^3^ = 10^6^ cm^3^; $\rho_{m}$ presents mountain density (g·cm^−3^); $UEV_{m}$ means the UEV of mountain (sej·g^−1^).

##### Climate regulation

According to the “United Nations Framework Convention on Climate Change” (UFNCCC), climate change is mainly manifested in global warming, acid rain and ozone destruction, of which global warming is the most urgent problem for human. Meanwhile, according to the data availability in the literature IPCC (2013), Roelfsema et al. (2018) and Goedkoop and Spriensma (2001), climate regulation service in this study mainly considers the global ecosystems as carbon sink to reduce the harm of climate change on human health and ecosystem quality, and the annual average carbon sequestration per unit area of the global ecosystem is applied to calculate climate regulation service here. Because of the availability of data, the greenhouse gases investigated in this study include CO_2_, CH_4_, NO_x_ and hydrofluorocarbon (HFC). The specific calculation method is as follows.

(Equation 25) $$Em_{cr1}=\sum \left(C_{ij}\cdot \frac{DALY_{gi}}{LT_{i}}\cdot S_{j}^{\prime }\cdot \tau_{H}\right)$$

(Equation 26) $$Em_{cr2}=\sum \left(C_{ij}\cdot \frac{PDF_{gi}}{LT_{i}}\cdot Em_{spj}\right)$$

where $Em_{cr1}$ means the emergy applied to reduce harms to human health resulting from climate regulation by ecosystems (sej·yr^−1^); $Em_{cr2}$ indicates the emergy needed to reduce harms to ecosystem quality brought by climate regulation by ecosystems (sej·yr^−1^); $C_{ij}$ is the *i-*th greenhouse gas sequestration in ecosystem *j* (kg·m^−2^·yr^−1^); $DALY_{gi}$ presents the DALY caused by greenhouse gas *i* (cap·yr·kg^−1^); $LT_{i}$ is the lifetime of greenhouse gas *i*; $\tau_{H}$ means the emergy per capita of human health inputs in case area (sej·cap^−1^); $S_{j}^{\prime }$ indicates the *j*th aquatic ecosystem’s area (ha);$\;PDF_{gi}$ presents the PDF of species resulting from greenhouse gas *i* (PDF·m^2^·yr·kg^−1^); $Em_{spj}$ is the emergy used to support species in ecosystem *j* (sej·yr^−1^), which is the local renewable resources (sej·yr^−1^) and can be calculated by Equation (4). The total value of climate regulation ($Em_{CR}$) for ecosystem is the sum of $Em_{cr1}$ and $Em_{cr2}$.

#### Total ES accounting principles

Because one ecological process can generate more than one ES, for example, both NPP and carbon sequestration are the products of photosynthesis, then biomass is the key source of soil organic matter. Therefore, to avoid double counting, this study establishes the summation principle of total ES, i.e. taking the maximum energy input of the same ecological process.

For forest, shrub and grassland ecosystems, the total ES are $Em_{ft}$, $Em_{st}$ and $Em_{gt}$ respectively and can be calculated as follows:

(Equation 27) $$Em_{t}=\sum \left(Max\left(Em_{NPP},\;Em_{CS},Em_{SB},Em_{GR},Em_{MR}\right),Em_{AP},Em_{SR},Em_{CR}\right)$$

For river ecosystems, runoff geopotential energy is one of the inputs to NPP service and also drives material transport. Therefore, the total river ecosystem service ($Em_{rt}$) of one region in this study does not include material transport service. Meanwhile, hydropower potential (n) is also partially driven by runoff geopotential energy. Assuming that the ratio of the distance from the hydropower station to the beginning of the river to the total length of the river in a given area is X, there are three cases for the location of hydropower station: (1) The start point of the river reach (X = 0); (2) the end point of the river reach (X = 1); (3) the location between the start point and the end point (0 < X < 1). For NPP service, there are also two cases: (a) runoff geopotential is the largest renewable resource; (b) rain chemical energy, wind energy or other forms of energy is the largest renewable resource. If it is case (a), the three cases of the hydropower station location should be considered, and the specific total river ES ($Em_{rt}$) is calculated as follows:

For case (a) and (1),

(Equation 28) $$Em_{rt}=\sum \left(MAX\left(Em_{NPP},\;Em_{CS},Em_{SBa},Em_{GR},Em_{MR}\right),\;Em_{WP},Em_{AP},Em_{HG},Em_{CR}\right)$$

For case (a) and (2),

(Equation 29) $$Em_{rt}=\sum (\left(1-x\right)\cdot MAX\left(Em_{NPP},\;Em_{CS},Em_{SB}a,Em_{GR},Em_{MR}\right),\;Em_{WP},Em_{AP},Em_{HG},Em_{CR})$$

For case (a) and (3),

(Equation 30) $$Em_{rt}=\sum \left(MAX\left(MAX\left(Em_{NPP},\;Em_{CS},Em_{SBa},Em_{GR},Em_{MR}\right),Em_{HG}\right),\;Em_{WP},Em_{AP},Em_{CR}\right)$$

For case (b),

(Equation 31) $$Em_{rt}=\sum \left(MAX\left(Em_{NPP},\;Em_{CS},Em_{SBa},Em_{GR},Em_{MR}\right),\;Em_{WP},Em_{AP},Em_{HG},Em_{CR}\right)$$

where all meanings of subtypes of ES are the same as the explanation in (Equation 3), (Equation 5), (Equation 6), (Equation 7), (Equation 8), (Equation 9), (Equation 10), (Equation 11), (Equation 12), (Equation 13), (Equation 14), (Equation 15), (Equation 16), (Equation 17), (Equation 18), (Equation 19), (Equation 20), (Equation 21), (Equation 22), (Equation 23), (Equation 24), (Equation 25), (Equation 26).

In terms of other aquatic ecosystems, the formula of total aquatic ES ($Em_{at}$) is as follows.

(Equation 32) $$Em_{at}=\sum \left(MAX\left(Em_{NPP},\;Em_{CS},Em_{SB},Em_{GR,}Em_{MR}\right),\;Em_{WP},Em_{AP},Em_{CR}\right)$$

The total ES in a study area is the sum of $Em_{ft}$, $Em_{st}$, $Em_{gt}$ and $Em_{at}$.

#### Attribution analysis method of changes in ES

##### General attribution analysis based on partial differential equation

Partial differential equations are a set of differential equations of multidimensional systems modeled as a function of several independent variables and their partial derivatives with respect to those variables (Jost, 2013). To our best knowledge, other than a study by Huang et al. (2019) where PDE was applied to identify the effect of precipitation, potential evapotranspiration and vegetation changes on runoff in the upper reaches of Xin’an River, PDE has not been yet used to account for the various factors contributing to changes in ES. Here are the general steps of using PDE for attribution analysis. Suppose Y is dependent variable; A, B, and C are independent variables, and they have following relationship:

(Equation 33) Y=A+B+C

Therefore, the change in Y in a given time, i.e., ΔY, can be written as the sum of the contributions of the three variables, expresses in the form of full differential:

(Equation 34) $$\Delta Y=\frac{\partial Y}{\partial \text{A}}\partial \text{A}+\frac{\partial Y}{\partial \text{B}}\partial \text{B}+\frac{\partial Y}{\partial \text{C}}\partial \text{C}$$

(Equation 35) $$\Delta Y=\left(\frac{\partial Y}{\partial \text{A}}\cdot \frac{A}{Y}\right)\cdot \frac{\Delta A}{A}\cdot Y+\left(\frac{\partial Y}{\partial \text{B}}\cdot \frac{B}{Y}\right)\cdot \frac{\Delta B}{B}\cdot Y+\left(\frac{\partial Y}{\partial \text{C}}\cdot \frac{C}{Y}\right)\cdot \frac{\Delta C}{C}\cdot Y+\delta$$

(Equation 36) $$\Delta Y=\varepsilon_{A}\cdot \frac{\Delta A}{A}\cdot Y+\varepsilon_{B}\cdot \frac{\Delta B}{B}\cdot Y+\varepsilon_{C}\cdot \frac{\Delta C}{C}\cdot Y+\delta$$

(Equation 37) $$\Delta Y=C_{r\_}A+C_{r\_}B+C_{r\_}C+\delta$$

where, δ is error; $C_{r\_}A$, $C_{r\_}B$ and $C_{r\_}C$ represent the contribution of changes in A, B and C to ΔY; $\varepsilon_{A}$, $\varepsilon_{B}$ and $\varepsilon_{C}$ are the elasticity coefficient of ΔY to A, B and C, which can be calculated by the partial differential expression of the three variables as follows: $\frac{\partial Y}{\partial \text{A}}$, $\frac{\partial Y}{\partial \text{B}}$ and $\frac{\partial Y}{\partial \text{C}}$.

Therefore, the contribution rate of A, B, C and δ to ΔY can be calculated as follows:

(Equation 38) $$R_{A}=\frac{C_{r\_}A}{\Delta Y}$$

(Equation 39) $$R_{B}=\frac{C_{r\_}B}{\Delta Y}$$

(Equation 40) $$R_{C}=\frac{C_{r\_}C}{\Delta Y}$$

(Equation 41) $$R_{\delta }=\frac{\text{δ}}{\Delta Y}$$

#### Attribution analysis techniques of changes of ES

Taking forest ecosystem as an example, the total forest ESs can be calculated as follows:

(Equation 42) $$T_{f}=\text{Max}\left(Em_{NPP},Em_{CS},Em_{SB},Em_{GR},Em_{MR}\right)+Em_{AP}+Em_{SR}+Em_{CR}$$

(Equation 43) $$T_{f}=\text{Max}R+Em_{AP}+Em_{SR}+Em_{CR}$$

(Equation 44) $$T_{f}=\text{MaxR}+\text{AC}\cdot \text{S}\cdot \text{DALY}\cdot \text{τ}+\text{AC}\cdot \text{PDF}\cdot \text{R}+\text{RM}\cdot \text{S}+\text{CS}\cdot \text{S}\cdot \text{DALY}\cdot \text{τ}$$

where, MaxR is the local renewable resource; AC represents air purification capacity of ecosystem (kg·ha^−1^·yr^−1^); S is the forest area in study area (m^2^); τ is the emergy of human health investment per capita (sej·cap^−1^); RM indicates soil retention amount (t·km^−2^·yr-1); CS refers to the greenhouse gas sequestration by ecosystem (kg·m^−2^·yr^−1^).

Because of AC, DALY, PDF, RM, CS are constants in this study, Equation (44) can be abbreviated as follows:

(Equation 45) $$T_{f}=\text{R}+k_{1}\cdot \text{S}\cdot \text{τ}+k_{2}\cdot \text{R}+k_{3}\cdot \text{S}+k_{4}\cdot \text{S}\cdot \text{τ}$$

(Equation 46) $$T_{f}=\left(1+k_{2}\right)\cdot R+\left(k_{1}+k_{4}\right)\cdot S\cdot \text{τ}+k_{3}$$

where, R= the maximum of the five services; $k_{1}=AC\cdot DALY$; $k_{2}=AC\cdot PDF$; $k_{3}=RM$; $k_{4}=CS\cdot DALY$.

Under such a circumstance, there are five cases to obtain $T_{f}$.

Case 1: $R=Em_{NPP}$;
Case 2: $R=Em_{CS}$;
Case 3: $R=Em_{SB}$;
Case 4: $R=Em_{GR}$;
Case 5: $R=Em_{MR}$.

According to the specific accounting techniques of $Em_{NPP}$, $Em_{CS}$, $Em_{SB}$, $Em_{GR}$ and $Em_{MR}$, stripping the constants in the accounting equations, we obtain the specific natural factors (*R*) in this study including precipitation, wind, elevation, NPP, biomass carbon density and ET, whose changes would cause changes in ES. *S*, i.e., ecosystem area, in this study is defined as the anthropogenic factor affecting changes in ES. τ is the emergy corresponding to the total health expenditure per capita in the region. Because the higher the medical expense reflects the greater attention to health care, thereby the greater significance degree of human attention to ecosystem service improvement. Therefore, τ is regarded as cognition degree driver in this study.

Taking case 5 as an example,

(Equation 47) $$T_{f}=\left(1+k_{2}\right)\cdot E\cdot S+\left(k_{1}+k_{4}\right)\cdot S\cdot \text{τ}+k_{3}\cdot \text{S}$$

where, E is the emergy needed by evapotranspiration of study area (sej·m^−2^·yr^−1^).

Therefore, $T_{f}$ is the function of E, τ and S, which are independent variables. The change in total forest ES, i.e., $\Delta T_{f}$, is contributed by these three variables, which can be expressed by the full differential equation:

(Equation 48) $$\Delta T_{f}=\frac{\partial T_{f}}{\partial \text{E}}\partial \text{E}+\frac{\partial T_{f}}{\partial \text{τ}}\partial \text{τ}+\frac{\partial T_{f}}{\partial \text{S}}\partial \text{S}$$

(Equation 49) $$\Delta T_{f}=\left(\frac{\partial T_{f}}{\partial \text{E}}\cdot \frac{E}{T_{f}}\right)\cdot \frac{\Delta E}{E}\cdot T_{f}+\left(\frac{\partial T_{f}}{\partial \text{τ}}\cdot \frac{\tau }{T_{f}}\right)\cdot \frac{\Delta \tau }{\tau }\cdot T_{f}+\left(\frac{\partial T_{f}}{\partial \text{S}}\cdot \frac{S}{T_{f}}\right)\cdot \frac{\Delta S}{S}\cdot T_{f}+\delta$$

(Equation 50) $$\Delta T_{f}=\varepsilon_{E}\cdot \frac{\Delta E}{E}\cdot T_{f}+\varepsilon_{\tau }\cdot \frac{\Delta \tau }{\tau }\cdot T_{f}+\varepsilon_{S}\cdot \frac{\Delta S}{S}\cdot T_{f}+\delta$$

(Equation 51) $$\Delta T_{f}=C_{r\_}E+C_{r\_}\tau +C_{r\_}S+\delta$$

where, δ is error; $C_{r\_}E$, $C_{r\_}\tau$ and $C_{r\_}S$ represent the contribution of changes in E, τ and S to $\Delta T_{f}$; $\varepsilon_{E}$, $\varepsilon_{\tau }$ and $\varepsilon_{S}$ are the elasticity coefficient of $\Delta T_{f}$ to E, τ and S, which can be calculated by the partial differential expression of the three variables as follows:

(Equation 52) $$\frac{\partial T_{f}}{\partial \text{E}}=\left(1+k_{2}\right)\cdot S$$

(Equation 53) $$\frac{\partial T_{f}}{\partial \text{τ}}=\left(k_{1}+k_{4}\right)\cdot S$$

(Equation 54) $$\frac{\partial T_{f}}{\partial \text{S}}=\left(1+k_{2}\right)\cdot E+\left(k_{1}+k_{4}\right)\cdot \tau +k_{3}$$

Therefore, the contribution rate of E, τ, S and δ to $\Delta T_{f}$ can be calculated as follows:

(Equation 55) $$R_{E}=\frac{C_{r\_}E}{\Delta T_{f}}$$

(Equation 56) $$R_{\tau }=\frac{C_{r\_}\tau }{\Delta T_{f}}$$

(Equation 57) $$R_{S}=\frac{C_{r\_}S}{\Delta T_{f}}$$

(Equation 58) $$R_{\delta }=\frac{\text{δ}}{\Delta T_{f}}$$

For all the cases, the contribution rate of R to $\Delta T_{f}$ is as follows:

(Equation 59) $$R_{fr}=\frac{C_{r\_}R}{\Delta T_{f}}$$

After obtaining the contribution rate of each ecosystem, the contribution rate of the total ES in one area are accounted as follows:

(Equation 60) $$\Delta \text{T}=\Delta T_{f}+\Delta T_{S}+\Delta T_{G1}+\Delta T_{G2}+\Delta T_{G3}+\Delta T_{W}+\Delta T_{L}+\Delta T_{R/P}+\Delta T_{R}$$

(Equation 61) $$\Delta \text{R}=\Delta T_{f}\cdot R_{fr}+\Delta T_{S}\cdot R_{sr}+\Delta T_{G1}\cdot R_{g1r}+\Delta T_{G2}\cdot R_{g2r}+\Delta T_{G3}\cdot R_{g3r}+\Delta T_{W}\cdot R_{wr}+\Delta T_{L}\cdot R_{lr}+\Delta T_{R/P}\cdot R_{r/pr}+\Delta T_{R}\cdot R_{rr}$$

(Equation 62) $$\Delta \text{τ}=\Delta T_{f}\cdot R_{f\tau }+\Delta T_{S}\cdot R_{s\tau }+\Delta T_{G1}\cdot R_{g1\tau }+\Delta T_{G2}\cdot R_{g2\tau }+\Delta T_{G3}\cdot R_{g3\tau }+\Delta T_{W}\cdot R_{w\tau }+\Delta T_{L}\cdot R_{l\tau }+\Delta T_{R/P}\cdot R_{r/p\tau }+\Delta T_{R}\cdot R_{r\tau }$$

(Equation 63) $$\Delta \text{S}=\Delta T_{f}\cdot R_{fs}+\Delta T_{S}\cdot R_{ss}+\Delta T_{G1}\cdot R_{g1s}+\Delta T_{G2}\cdot R_{g2s}+\Delta T_{G3}\cdot R_{g3s}+\Delta T_{W}\cdot R_{ws}+\Delta T_{L}\cdot R_{ls}+\Delta T_{R/P}\cdot R_{r/ps}+\Delta T_{R}\cdot R_{rs}$$

(Equation 64) $$R_{R}=\frac{\Delta R}{\Delta T}$$

(Equation 65) $$R_{\tau }=\frac{\Delta \tau }{\Delta T}$$

(Equation 66) $$R_{S}=\frac{\Delta S}{\Delta T}$$

(Equation 67) $$\delta =1-\sum \left(R_{R},R_{\tau },R_{S}\right)$$

where, ΔT, $\Delta T_{f}$, $\Delta T_{S}$, $\Delta T_{G1}$, $\Delta T_{G2}$, $\Delta T_{G3}$, $\Delta T_{W}$, $\Delta T_{L}$, $\Delta T_{R/P}$ and $\Delta T_{R}$ are the changes in total, forest, shrub, high cover grassland, medium cover grassland, low cover grassland, wetland, lake, reservoir/pond, and river ES between different times respectively. $R_{fr}$, $R_{sr}$, $R_{g1r}$, $R_{g2r}$, $R_{g3r}$, $R_{wr}$, $R_{lr}$, $R_{r/pr}$, $R_{rr}$ are the contribution rate of natural factors to the changes in total, forest, shrub, high cover grassland, medium cover grassland, low cover grassland, wetland, lake, reservoir/pond, and river ES respectively. $R_{f\tau }$, $R_{s\tau }$, $R_{g1\tau }$, $R_{g2\tau }$, $R_{g3\tau }$, $R_{w\tau }$, $R_{l\tau }$, $R_{r/p\tau }$ and $R_{r\tau }$ are the contribution rate of cognition degree drivers to the changes in total, forest, shrub, high cover grassland, medium cover grassland, low cover grassland, wetland, lake, reservoir/pond, and river ES respectively. $R_{fs}$, $R_{ss}$, $R_{g1s}$, $R_{g2s}$, $R_{g3s}$, $R_{ws}$, $R_{ls}$, $R_{r/ps}$ and $R_{rs}$ are the contribution rate of ecosystem area to the changes in total, forest, shrub, high cover grassland, medium cover grassland, low cover grassland, wetland, lake, reservoir pond, and river ES respectively. ΔR, Δτ and ΔS are the changes in R, τ and S in different periods. δ is the contribution rate of error to the change in total ES.

### Quantification and statistical analysis

Analyses and plots were performed with Microsoft Excel, PowerPoint, SPSS, and ArcGIS.

## Data Availability

• All data reported in this paper will be shared by the lead contact on request. • This paper does not report original code. • Any additional information required to reanalyze the data reported in this paper is available from the lead contact on request.

## Supporting Citations

The following reference appears in the Supplemental Information: Xu et al. (2018b); Hainan Water Authority (2016); Henan Province Bureau of Statistics (2016); Intergovernmental Science-Policy Platform on Biodiversity and Ecosystem Services et al. (2019); Shandong Province Bureau of Statistics (2016); UK National Ecosystem Assessment (2014).

## Supplemental information

Document S1. Tables S1–S3, S5–S9, S11–S13 and Figures S1 and S2

Table S4. Change rate of China’s ecosystem services (ES) in 2000–2020, related to Figure 1

Table S10. The LUCC transfer matrix of Shandong, Jilin and Heilongjiang Provinces from 2000 to 2020, related to Figure 2
