# Supplementary material for: Hidden challenges behind ecosystem services improvement claims
Source: iScience. 2022 Aug 13;25(9):104928. doi: 10.1016/j.isci.2022.104928 (PMC9440298; doi:10.1016/j.isci.2022.104928)
Supplement: Document S1. Tables S1–S3, S5–S9, S11–S13 and Figures S1 and S2 [file mmc1.pdf]

iScience, Volume *25*

Supplemental information

Hidden challenges behind ecosystem services improvement claims

Qing Yang, Gengyuan Liu, Linyu Xu, Sergio Ulgiati, Marco Casazza, Yan Hao, Zhongming Lu, Xiaoya Deng, and Zhifeng Yang

Supplemental Information

Supplemental figures and tables

Supplemental tables

Table S1. Ecosystem classification based on China's land use remote sensing data, Related to STAR Methods.

Code I	Class I	Code II	Class II
2	Woodland	21	Forest
		22	Shrub
3	Grassland	31	High coverage grassland
		32	Medium coverage grassland
		33	Low coverage grassland
4	Aquatic ecosystems	41	River
		42	Lake
		43	Reservoir/ponds
6	Unused land	64	Wetlands

Source: reference Xu et al. (2018b).

Table S2. Ecosystems and their services investigated in this study, Related to STAR Methods.

Ecosystem services	Forest	Shrub	HCG	MCG	LCG	River	Lake	R/P	Wetland
**NPP**	√	√	√	√	√	√	√	√	√
**Carbon sequestration**	√	√	√	√	√	√	√	√	√
**Soil building**	√	√	√	√	√				
**Groundwater recharge**	√	√	√	√	√	√	√	√	√
**Air purification**	√	√	√	√	√				
**Water purification**						√	√	√	√
**Materials transport**						√			
**Soil retention**	√	√	√	√	√				
**Hydropower potential (n)**						√			
**Microclimate regulation**	√	√	√	√	√	√	√	√	√
**Climate regulation**	√	√	√	√	√	√	√	√	√

" √ " means the ecosystems have their corresponding services. Blank space indicates the ecosystems do not have the corresponding services. Hydropower potential (n) means hydropower potential in this study only includes nature's contribution, such as precipitation and elevation difference, while does not include humans' inputs. HCG, MCG and LCG means high, moderate and low coverage grassland respectively; R/P: Reservoir or pond.

Table S3. Comparison of main driver systems of changes in ecosystem services, Related to Figure 2 and Table 2.

Driver classification	MA (2005)	IPBES (2019)	UK NEA (2014)	This study
Direct drivers	Climate variability and change	Climate change	Climate variability and change	Precipitation, evapotranspiration, solar radiation, wind speed
	Land conversion	Land-use change	Habitat change	Land-use change
	Biological invasions and diseases	Invasive species	Invasive species	
	Air pollution emissions, Use of Nitrogen fertilizer and Nitrogen loads to rivers and coastal marine systems	Pollution	Pollution and nutrient enrichment	
	Plant nutrient use	Natural resource use and exploitation	Overexploitation of resources	
Indirect drivers	Demographic drivers	Demographic trend	Demographic drivers	
	Economic drivers: consumption, production, and globalization		Economic drivers	Total health expenses
	Sociopolitical drivers	Socio-economic trend, government	Sociopolitical drivers	
	Cultural and religious drivers	Culture	Cultural and behavioral drivers	
	Science and technology drivers	Technological innovation	Scientific and technological drivers	

Note: The direct drivers of ES changes include land cover changes, overexploitation and pollution, which clearly affect ecosystem services by changing their natural processes. The subtypes of indirect drivers are economic, social, demographic, political, cultural and technological, which act more diffusely by revising one or more direct drivers (IPBES, 2019; MA, 2005). Most direct or indirect driver terms are applied synonymously and interchangeably. For example, nitrogen fertilizer and nitrogen load to rivers and coastal marine systems used in MA (2005) are same as nutrient enrichment used in UK NEA (2014). Similarly, the sub-categories of drivers in other studies can basically correspond to the sub-categories in this study in Table S3.

Table S5. The rank of the change rate of China's ecosystem services from 2000 to 2020, Related to Figure 2.

Province	Change rate
Tibet	49%
Chongqing	47%
Ningxia	41%
Liaoning	40%
Beijing	38%
Shanxi	31%
Hubei	31%
Shaanxi	31%
Jilin	31%
Inner Mongolia	30%
Tianjin	28%
Qinghai	24%
Gansu	24%
Hebei	21%
Hunan	20%
Guizhou	19%
Anhui	18%
Guangxi	16%
Xinjiang	16%
Sichuan	16%
Heilongjiang	15%
Jiangxi	15%
Zhejiang	14%
Jiangsu	10%
Guangdong	9%
Yunnan	4%
Shandong	-1%
Hainan	-4%
Henan	-6%
Fujian	-8%
Shanghai	-15%

Table S6. The contribution rate of R, τ, S and δ to the changes in China's ecosystem services during 2000-2020, Related to Figure 2.

Province	Factors				Province area ($10^4 km^2$)
	R	τ	S	δ	
**Provinces with ES increase**					
Yunnan	-147%	36%	208%	2%	38.33
Hebei	-30%	6%	121%	3%	18.77
Liaoning	-7%	7%	110%	-10%	14.59
Jilin	2%	6%	92%	-1%	18.74
Inner Mongolia	-2%	10%	91%	0%	118.3
Guangdong	-11%	19%	91%	2%	18
Jiangsu	-12%	32%	85%	-5%	10.26
Heilongjiang	5%	8%	79%	9%	47.3
Shaanxi	15%	3%	76%	6%	20.56
Xinjiang	5%	23%	76%	-3%	166
Tibet	13%	12%	71%	4%	122.8
Tianjin	-1%	25%	67%	9%	1.13
Jiangxi	23%	8%	66%	2%	16.7
Qinghai	39%	8%	64%	-11%	72.23
Guizhou	27%	9%	57%	7%	17.6
Shanxi	21%	17%	54%	8%	15.63
Anhui	23%	11%	53%	13%	13.97
Zhejiang	30%	23%	44%	3%	10.2
Chongqing	49%	2%	45%	3%	8.23
Gansu	55%	2%	52%	-8%	45.44
Beijing	46%	22%	39%	-8%	1.68
Sichuan	65%	3%	32%	1%	48.14
Hunan	70%	4%	25%	1%	21.18
Ningxia	55%	2%	20%	23%	6.64
Hubei	89%	1%	9%	1%	18.59
Guangxi	89%	5%	6%	0%	23.6
**Provinces with ES decrease**					
Shanghai	13%	72%	-163%	-21%	0.63
Shandong	-280%	157%	-155%	177%	15.38
Hainan	-149%	192%	-101%	-42%	3.4
Fujian	-141%	9%	26%	6%	12.13
Henan	-105%	3%	46%	-45%	16.7
**Total**	**37%**	**8%**	**55%**	**1%**	

R: Natural factors (such as precipitation, evapotranspiration, etc.); τ: Cognition degree driver (The significance degree of human attention to ecosystem services improvement); S: Human factor (land use change); δ: Errors.

Table S7. The rank of total health expenditure per capita of each province in China in 2020[a], Related to Figure 2.

Province	Total health expenditure per capita
Beijing	10184
Shanghai	8611
Tianjin	5545
Zhejiang	4926
Jiangsu	4585
Qinghai	4477
Xinjiang	4379
Ningxia	4343
Tibet	4190
Guangdong	4071
Inner Mongolia	3987
Shaanxi	3980
Hainan	3955
Chongqing	3803
Jilin	3726
Liaoning	3675
Hubei	3675
Sichuan	3663
Fujian	3571
Heilongjiang	3557
Shandong	3554
Yunnan	3130
Hunan	3113
Gansu	3082
Shanxi	2925
Hebei	2908
Guizhou	2900
Anhui	2866
Jiangxi	2703
Henan	2519
Guangxi	2462

Unit: 10,000yuan per capita; data source: China Health and Family Planning Statistical Yearbook in 2020 (http://tongji.cnki.net/kns55/Navi/YearBook.aspx?id=N2017010032&floor=1). a: due to the lack of data in 2020 and 2019, we applied the data of total health expenditure per capita in 2018 to replace the data in 2020.

Table S8. The annual average precipitation of Henan, Shandong and Hainan Provinces, Related to Figure 2.

Year	Henan	Shandong	Hainan	Fujian
2000	996.5	661.9	2170.4	1883.6
2001	533.0	577.8	-	-
2002	882.5	417.4	-	-
2003	1182.5	887.5	-	-
2004	806.0	787.0	-	-
2005	988.0	827.4	1756.5	1816.33
2006	825.0	542.5	-	-
2007	927.5	845.5	1690.4	-
2008	875.0	749.4	2095.2	-
2009	737.0	685.0	2275.0	-
2010	1032.0	701.8	2251.8	1961.31
2011	751.0	735.4	2273.3	-
2012	639.0	623.9	1939.9	-
2013	544.9	727.9	2393.7	-
2014	696.1	531.4	1993.0	-
2015	694.9	604.4	1403.5	1934.7
2020	874.3	838.1	1641.1	1439.1
Change rate from 2000 to 2020	-12.26%	26.62%	-24.39%	-23.60

Date Source: reference HPBS (2016); SPBS (2016); HWA (2016); MWRPRC (2020); - means no data. The unit of precipitation is mm.

Table S9. The ecosystem areas (m^2) of Shandong Province and the change rate from 2000 to 2020, Related to Figure 2.

Ecosystems	Ecosystem area			Change rate
	2000	2015	2020	2000-2020
Forest	4.17E+09	4.45E+09	4.65E+09	12%
Shrub	1.34E+09	1.34E+09	1.33E+09	-1%
HCG	2.85E+09	2.08E+09	2.15E+09	-25%
MCG	4.69E+09	3.31E+09	3.00E+09	-36%
LCG	1.96E+09	1.24E+09	1.24E+09	-37%
Wetland	1.26E+08	2.95E+08	3.02E+08	140%
Lake	8.34E+08	7.26E+08	7.25E+08	-13%
R/P	1.93E+09	4.47E+09	4.02E+09	108%
River	1.05E+09	1.22E+09	1.23E+09	17%
**Total**	**1.90E+10**	**1.91E+10**	**1.86E+10**	**-2%**

HCG, MCG and LCG: High, moderate and low coverage grassland; R/P: Reservoir or pond.

Table S11. The shrub ecosystem area (m^2) in Jilin and Heilongjiang provinces, Related to Figure 2.

Province	2000	2020	Change rate
Jilin	2.34E+09	1.93E+09	-17%
Heilongjiang	1.98E+10	6.32E+09	-68%

Table S12. The contribution rate of nine ecosystems to the changes in China's ecosystem services from 2000 to 2020, Related to Figure 3.

Ecosystem types	Provinces	Forest	Shrub	HCG	MCG	LCG	Wetland	Lake	R/P	River
**Provinces with ES increase**										
Forest	Heilongjiang	134%	-41%	-4%	-1%	0%	6%	1%	0%	5%
	Yunnan	106%	62%	8%	11%	3%	0%	0%	1%	-91%
	Guangdong	103%	1%	3%	0%	0%	0%	0%	-5%	-3%
	Jilin	100%	-2%	2%	-2%	0%	-1%	0%	0%	3%
	Liaoning	96%	4%	2%	-7%	-1%	0%	0%	3%	4%
	Jiangxi	87%	3%	2%	0%	0%	-1%	-3%	0%	13%
	Zhejiang	76%	2%	2%	0%	0%	0%	0%	1%	19%
	Hebei	73%	26%	26%	4%	0%	0%	0%	6%	-36%
	Tianjin	66%	3%	-3%	-7%	0%	0%	2%	40%	-1%
	Chongqing	65%	-10%	1%	-4%	0%	0%	0%	0%	47%
	Beijing	64%	-1%	2%	0%	0%	0%	0%	0%	34%
	Tibet	62%	23%	-30%	8%	22%	0%	10%	0%	3%
	Anhui	60%	25%	13%	0%	0%	0%	1%	1%	-1%
	Inner Mongolia	42%	6%	27%	17%	7%	0%	0%	0%	0%
	Shanxi	37%	19%	5%	4%	12%	0%	0%	0%	23%
	Shaanxi	24%	20%	14%	23%	9%	0%	0%	0%	9%
Shrub	Guizhou	26%	36%	1%	14%	-1%	0%	0%	0%	23%
MCG	Xinjiang	-14%	-7%	64%	12%	30%	0%	12%	0%	1%
River	Hubei	8%	4%	0%	0%	0%	0%	0%	0%	87%
	Guangxi	8%	7%	3%	0%	0%	0%	0%	0%	82%
	Hunan	33%	5%	0%	0%	0%	0%	-1%	0%	62%
	Sichuan	22%	9%	4%	9%	1%	0%	0%	0%	54%
	Ningxia	1%	6%	2%	15%	21%	0%	0%	1%	54%
	Gansu	7%	7%	5%	14%	15%	0%	0%	0%	51%
	Qinghai	2%	10%	6%	22%	21%	-1%	5%	2%	34%
R/P	Jiangsu	18%	-6%	-4%	0%	0%	0%	25%	57%	10%
**Provinces with ES decrease**										
HCG	Shandong	633%	12%	-358%	-757%	-276%	35%	-10%	574%	47%
River	Shanghai	0%	0%	4%	0%	0%	0%	0.13%	-24%	-130%
	Hainan	-27%	-33%	-8%	-1%	0%	0%	0%	8%	-39%
	Fujian [a]	13%	7%	2%	0%	-2%	0.03%	-0.03%	1%	-121%
	Henan [a]	33%	6%	3%	-1%	0%	0%	0%	2%	-143%
	**Total**	**39%**	**9%**	**3%**	**7%**	**6%**	**0%**	**2%**	**0%**	**33%**

HCG, MCG, LCG: high, medium and low coverage grassland; R/P: Reservoir or pond. a: Ecosystem services declined in Fujian and Henan mainly resulted from the worse of natural factors.

Table S13. The contribution rate of R, τ, S and δ to the changes of forest, shrub and HCG ecosystem services in the forest or shrub or grassland-dominated provinces, Related to Figure 3.

Dominant ecosystem	Provinces	R	τ	S	δ
Forest	Liaoning	-5%	3%	106%	-5%
	Yunnan	-20%	17%	102%	0%
	Hebei	1%	4%	95%	0%
	Jilin	0%	6%	92%	2%
	Heilongjiang	0%	5%	90%	5%
	Chongqing	2%	1%	90%	7%
	Tianjin	0%	6%	88%	6%
	Guangdong	-4%	16%	86%	1%
	Inner Mongolia	-1%	7%	85%	8%
	Shaanxi	7%	7%	84%	3%
	Jiangxi	10%	8%	79%	3%
	Tibet	3%	9%	78%	11%
	Beijing	-1%	24%	68%	9%
	Shanxi	1%	24%	63%	12%
	Anhui	30%	13%	53%	4%
	Zhejiang	17%	29%	51%	3%
Shrub	Guizhou	5%	13%	77%	5%
HCG	Xinjiang	4%	5%	92%	-2%

R: Natural factors (such as precipitation, evapotranspiration, etc.); τ: Cognition degree driver (The significance degree of human attention to ecosystem services improvement); S: Human factor (land use change); δ: Errors; HCG: high coverage grassland.

Supplemental figures

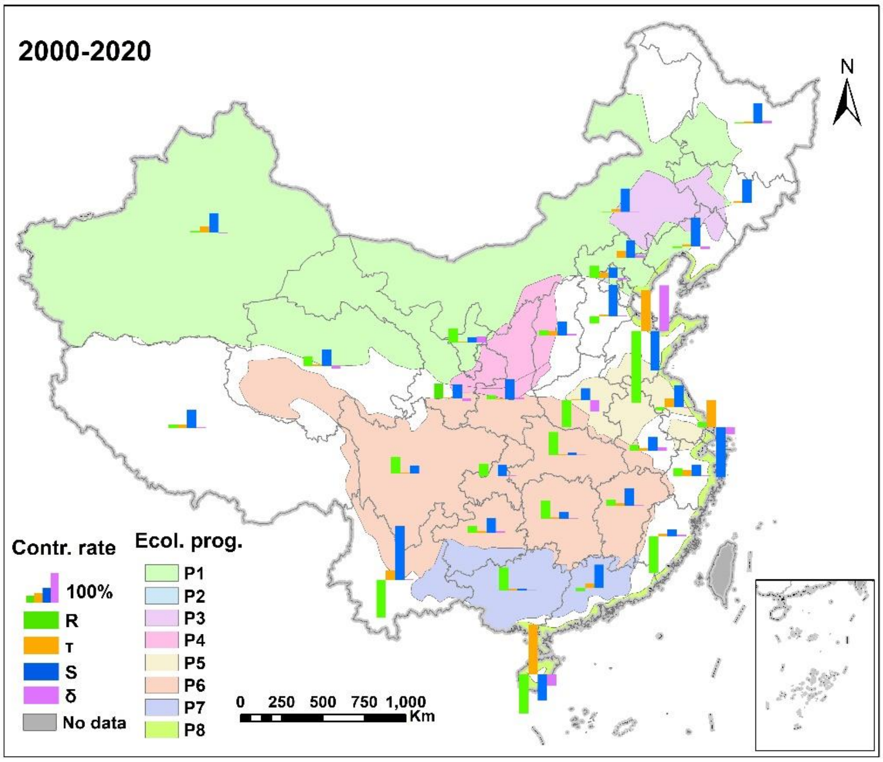

Figure S1. The contribution rate of the changes in ESs and China's ecological programmes, Related to Figure 2. (Key of symbols and abbreviations: Contr. Rate: Contribution rate; R: Natural drivers (such as precipitation, evapotranspiration, etc.); τ: Cognition degree driver (The significance degree of human attention to ecosystem services improvement); S: Human driver (land use change); δ: Errors. Ecol. prog.: ecological programs; P1: Three-north shelterbelt program; P2: Afforestation program for Taihang mountain; P3: Shelterbelt program for Liaohe river; P4: Shelterbelt program for middle reaches of the Yellow river; P5: Shelterbelt program for Huaihe river and Taihu lake; P6: Shelterbelt program for upper and middle reaches of Yangtze river; P7: Shelterbelt program for Pearl river; P8: Coastal shelterbelt program)

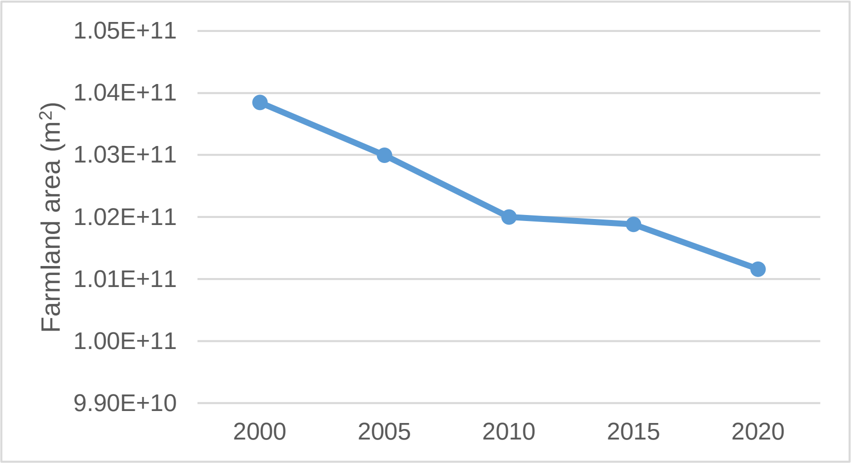

Figure S2. Farmland area of Shandong Province during 2000-2020, Related to Figure 2.

Data Source: reference Xu et al. (2018b)
